# Discrete spatio-temporal regulation of tyrosine phosphorylation directs influenza A virus M1 protein towards its function in virion assembly

**DOI:** 10.1371/journal.ppat.1008775

**Published:** 2020-08-31

**Authors:** Angeles Mecate-Zambrano, Swathi Sukumar, Guiscard Seebohm, Kevin Ciminski, André Schreiber, Darisuren Anhlan, Lilo Greune, Ludmilla Wixler, Stephanie Grothe, Nora Caroline Stein, M. Alexander Schmidt, Klaus Langer, Martin Schwemmle, Tianlai Shi, Stephan Ludwig, Yvonne Boergeling

**Affiliations:** 1 Institute of Virology Muenster, University of Muenster, Muenster, Germany; 2 Cells in Motion Interfaculty Centre (CiM), University of Muenster, Muenster, Germany; 3 Institute for Genetics of Heart Diseases (IfGH), Department of Cardiovascular Medicine, University Hospital Muenster, Muenster, Germany; 4 Institute of Virology, Medical Center–University of Freiburg, Freiburg, Germany; 5 Faculty of Medicine, University of Freiburg, Freiburg, Germany; 6 Institute of Infectiology, Center for Molecular Biology of Inflammation (ZMBE), University of Muenster, Muenster, Germany; 7 Institute of Pharmaceutical Technology and Biopharmacy, University of Muenster, Muenster, Germany; 8 Immunology, Inflammation and Infectious Diseases (I3) DTA, Roche Pharma Research and Early Development, Roche Innovation Center Basel, Basel, Switzerland; University of Texas Southwestern Medical Center, UNITED STATES

## Abstract

Small RNA viruses only have a very limited coding capacity, thus most viral proteins have evolved to fulfill multiple functions. The highly conserved matrix protein 1 (M1) of influenza A viruses is a prime example for such a multifunctional protein, as it acts as a master regulator of virus replication whose different functions have to be tightly regulated. The underlying mechanisms, however, are still incompletely understood. Increasing evidence points towards an involvement of posttranslational modifications in the spatio-temporal regulation of M1 functions. Here, we analyzed the role of M1 tyrosine phosphorylation in genuine infection by using recombinant viruses expressing M1 phosphomutants. Presence of M1 Y132A led to significantly decreased viral replication compared to wildtype and M1 Y10F. Characterization of phosphorylation dynamics by mass spectrometry revealed the presence of Y132 phosphorylation in M1 incorporated into virions that is most likely mediated by membrane-associated Janus kinases late upon infection. Molecular dynamics simulations unraveled a potential phosphorylation-induced exposure of the positively charged linker domain between helices 4 and 5, supposably acting as interaction platform during viral assembly. Consistently, M1 Y132A showed a defect in lipid raft localization due to reduced interaction with viral HA protein resulting in a diminished structural stability of viral progeny and the formation of filamentous particles. Importantly, reduced M1-RNA binding affinity resulted in an inefficient viral genome incorporation and the production of non-infectious virions that interferes with virus pathogenicity in mice. This study advances our understanding of the importance of dynamic phosphorylation as a so far underestimated level of regulation of multifunctional viral proteins and emphasizes the potential feasibility of targeting posttranslational modifications of M1 as a novel antiviral intervention.

## Author summary

Due to limited genome capacity, viral proteins often fulfill multiple functions during viral replication. Matrix proteins of enveloped viruses have to be intrinsically multifunctional, since they confer integrity of the virion, but also function in disintegration during virus entry. The highly conserved influenza A virus matrix protein 1 (M1) is a prototype for such a multifunctional protein, however, the underlying mechanisms that regulate its functional switches are only scarcely understood. Here, we unraveled M1 phosphorylation at tyrosine 132 as a spatio-temporal regulatory mechanism that potentially induces structural diversification and is needed for efficient viral genome incorporation during assembly. The site is most likely phosphorylated by cellular membrane-associated Janus kinases late in infection, suggesting that these kinases might serve as novel cellular targets for antiviral intervention.

## Introduction

Due to limited genome capacity, viruses strongly rely on host cell functions to replicate efficiently. Viruses capture and modify the host cell machinery for their own needs. Furthermore, viral proteins often possess multiple functions during replication and this multifunctionality needs to be tightly regulated. Possible regulatory means include posttranslational modifications by cellular enzymes, such as ubiquitous phosphorylation by kinases. This allows for dexterous tuning of viral protein functions in a spatio-temporal manner. In the course of advancements in mass spectrometry approaches, phosphorylation of viral proteins of different viruses has been extensively studied [1]. However, despite increasing knowledge of phosphorylation sites in specific viral proteins, the biological meaning of most of these modifications is still far from being resolved.

Matrix proteins of enveloped viruses have to be intrinsically multifunctional, since they do not only confer integrity of the virion supporting the fragile lipid envelope, but also have to be eligible for disintegration during the process of uncoating [2]. Matrix protein 1 (M1) of influenza A viruses (IAV) is a paradigm of such viral proteins. IAV M1 provides an interaction platform that is not only important for virion morphology and stabilization of the two major viral surface glycoproteins, but also keeps the segmented genome of eight ribonucleoprotein complexes (vRNPs) attached in the core of the virion, finally orchestrating efficient assembly and budding [3]. Noteworthy, these interactions need to be dispersed during the process of uncoating, where the vRNPs are released into the cytoplasm [4,5]. In addition, M1 shuttles into the nucleus, inhibiting viral polymerase activity of newly synthesized vRNPs, and guides nuclear export [6–8]. These different functions have been shown to involve multiple conformational transitions in M1 structures [2], while the underlying cooperative molecular mechanisms still remain elusive.

Several phosphorylation sites of IAV proteins have been identified [9–11]. However, deeper understanding how these modifications are involved in the regulation of the different steps of the IAV life cycle is missing. So far, only one of the identified M1 phosphorylation sites has been functionally studied by overexpression approaches. It was suggested that phosphorylation of tyrosine 132 (Y132) is needed for efficient M1 import into the nucleus by mediating importin α interaction [12]. However, it is frequently observed that overexpression of viral proteins can lead to artificial behavior and does not necessarily reflect functions in genuine infection. In particular, M1 functions have been shown to strongly depend on expression levels [13,14] and, furthermore, the role of spatio-temporal regulation of M1 functions by phosphorylation in genuine infection has not been addressed so far. Unraveling these mechanisms and functions is key for the design of antiviral strategies, as targeting of multifunctional M1 might provide the opportunity to block infection at various levels of virus replication.

Here, we analyzed the function of M1 tyrosine phosphorylation in genuine infection and revealed an essential role of Y132 phosphorylation that is most likely mediated by Janus kinases in late stages of virus replication. We found that phosphorylation potentially induces structural changes and is involved in M1 lipid raft localization and viral genome incorporation.

## Results

### Phosphorylation of M1 Y132 is needed for efficient virus replication

**Fig 1:**
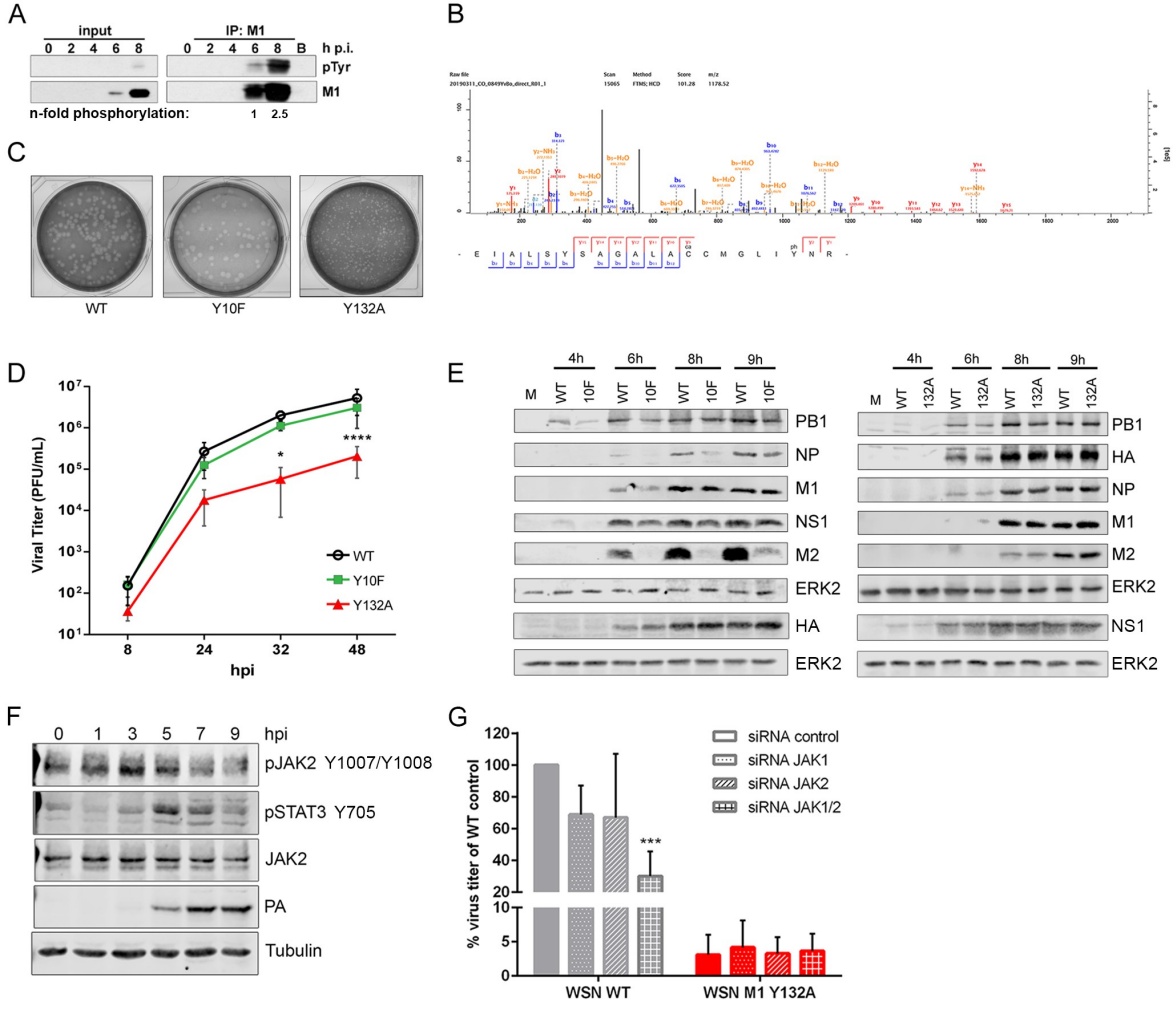
Phosphorylation of M1 Y132 late in the infection cycle is needed for efficient virus replication. **A)** M1 was immunoprecipitated with M1-specific antibodies from WSN-infected A549 cells (5 MOI; 2, 4, 6, 8 hpi) followed by analysis of M1 tyrosine phosphorylation in western blot by using pY antibodies. IP efficiency was analyzed by detection of M1. Blots are representative of two independent experiments. N-fold increase in pY M1 was analyzed by densitometric analysis of respective band intensities of pY M1 and total M1. Phosphorylation intensities were normalized to respective M1 amounts and pY detected 6 hpi was arbitrarily set to 1. **B)** MS/MS spectrum of the tryptic peptide EIALSYSAGALACCMGLIYNR of M1 isolated from WSN virus particles showing phosphorylation at Y132. LC-MS/MS analyses were performed on a Proxeon Easy-nLC coupled to a QExactice mass spectrometer. The MS data were processed using MaxQuant software (version 1.5.2.8. with integrated Andromeda search engine). **C)** Plaque phenotypes of WSN WT (left), M1 Y10F (middle) and M1 Y132A (right). Dilutions from standard plaque assays were stained with neutral red. **D)** A549 cells were infected with 0.01 MOI WSN WT, M1 Y10F or M1 Y132A. Virus-containing supernatants were harvested 8, 24, 32 and 48 hpi. Titers were determined by standard plaque assays and are depicted as mean (±SD) of three independent experiments. Statistical significance was analyzed by two-way Anova followed by Dunett’s multiple comparison test (*p≤0.05, ****p≤0.0001). **E)** A549 cells were infected with 1 MOI WSN WT, M1 Y10F or M1 Y132A and expression of viral proteins PB1, NP, HA, M1, NS1 and M2 was analyzed 4, 6, 8 and 9 hpi. ERK2 expression served as loading control. Blots are representative of three independent experiments. **F)** A549 cells were infected with 5 MOI WSN WT and activation of JAK2 and STAT3 was analyzed by detection of phosphorylation of Y1007/Y1008 (JAK2) and Y705 (STAT3), respectively. Expression of viral PA was analyzed to confirm efficient infection. JAK2 and tubulin expression served as loading controls. Blots are representative of three independent experiments. **G)** A549 cells were transfected with JAK1-, JAK2- or a combination of specific siRNAs for 48 h and were subsequently infected with 0.1 MOI WSN WT or M1 Y132A. Virus-containing supernatants were harvested 8 hpi and viral titers were determined by standard plaque assays and are depicted as mean (±SD) of three independent experiments. Statistical significance was analyzed by two-way Anova followed by Dunett’s multiple comparisons test (***p≤0.001).

IAV M1 is phosphorylated at serine, threonine and tyrosine residues [9,11]. Here, we particularly focused on dynamics of M1 tyrosine phosphorylation (pY) during replication. pY western blots revealed an 2.5-fold increase in M1 pY over time, being most prominent at late stages of viral replication (Fig 1A). Mass spectrometry of overexpressed GST-tagged M1 confirmed phosphorylation of two M1 pY sites already identified, Y10 and Y132 [9,12]. Of note, respective peptides of both sites were not detected 7 hours post infection (hpi), but were particularly phosphorylated 9 hpi. Compared to a dephosphorylated sample, there was repeated spectral evidence at low intensity for several phosphorylated tryptic peptides from the Y132 region (S1A Fig), while software assigned Y10 phosphorylation could not definitely be confirmed by manual inspection. Low stoichiometric amounts of specific phosphorylation patterns most likely reflect the presence of diverse functional M1 subpopulations. Interestingly, analysis for the presence of pY in endogenously expressed M1 incorporated into released viral particles provided evidence of phosphorylated Y132 (Fig 1B). These results reflect the previously observed increase in M1 tyrosine phosphorylation late during the viral replication cycle (Fig 1A).

Although phosphorylation of Y10 was previously identified, it was not functionally analyzed [9]. Phosphorylation of M1 Y132 was suggested to be crucial for virus fitness, since the authors failed to rescue respective virus mutants [12]. Here, WSN mutants carrying non-phosphorylatable amino acids (aa) at position 10 or 132 (Y10F, Y132A) were successfully generated, allowing to study these sites in genuine infection. Phospho-mimetic substitution mutants (Y10D, Y132D) could not be rescued, indicating that these mutations, providing a constant negative charge similar to phosphorylation, are not tolerated in virus replication.

Plaques of WSN M1 Y132A were significantly smaller than that of wildtype (WT) and WSN M1 Y10F (Fig 1C, S1B Fig). In multicycle replication studies, WSN M1 Y132A titers were reduced up to one order of a magnitude, while WSN M1 Y10F showed only marginal differences compared to WT (Fig 1D). Furthermore, expression levels of viral proteins were similar for WSN M1 Y10F when compared to WT (Fig 1E, left panel, S1C Fig), with exception of M2 expression. Since the nucleotide changes leading to Y10F mutation are localized directly next to the donor splice site resulting in a change in the reported consensus (AACGUACGUUC to AACGUUCGUUC) of the splicing signal [15], reduced M2 expression might be due to aberrant splicing. This, however, does not affect expression of other viral proteins or viral replication, overall highlighting that M1 Y10 phosphorylation is not needed for efficient viral replication. Regarding WSN M1 Y132A infection, viral protein expression was not altered (Fig 1E, right panel), however, there was a clear impact on viral titers (Fig 1D) suggesting that M1 Y132 phosphorylation affects later stages of replication, which correlates to the tyrosine phosphorylation kinetic and presence of pY132 in M1 incorporated into released viral particles (Fig 1A and 1B).

Phosphorylation of M1 Y132 was reported to be catalyzed by Janus kinases 1/2 (JAK1/2) [12]. While JAK1 shows only marginal expression in A549 cells, there was a clear activation of JAK2 (phosphorylation of JAK2 at Y1007/Y1008) and its downstream target signal transducer and activator of transcription 3 (STAT3, phosphorylation at Y705) detectable upon WSN infection (Fig 1F). JAK2 has already been described to be a host factor needed for efficient replication but the underlying mechanism is still unclear [16,17]. To functionally link JAK2 dependency with a potential JAK-mediated M1 Y132 phosphorylation, JAK1/2 were knocked-down and viral replication efficiencies were analyzed for WSN WT as well as WSN M1 Y132A. Virus titers of WSN WT were significantly decreased by approximately 70% in absence of both JAKs indicating a compensatory function of these kinases. In contrast, WSN M1 Y132A was not affected (Fig 1G). To verify a direct phosphorylation of M1 by JAK2, *in vitro* kinase assays were performed, clearly demonstrating M1 tyrosine phosphorylation mediated by JAK2 (S1D Fig). However, no differences in phosphorylation intensities were observed in presence of M1 Y132A or Y132F compared to WT. Nevertheless, mass spectrometry-based identification of pY sites in WT M1 provided spectral evidence of phosphorylated tryptic peptides from the Y132 region. These results indicate that receptor-associated JAKs seem to catalyze M1 Y132 phosphorylation, most likely when M1 reaches the plasma membrane late upon infection to initiate virus assembly and budding.

### Molecular dynamic simulation indicates that phosphorylation of M1 Y132 may induces structural repositioning of the linker domain between helices 4 and 5

**Fig 2:**
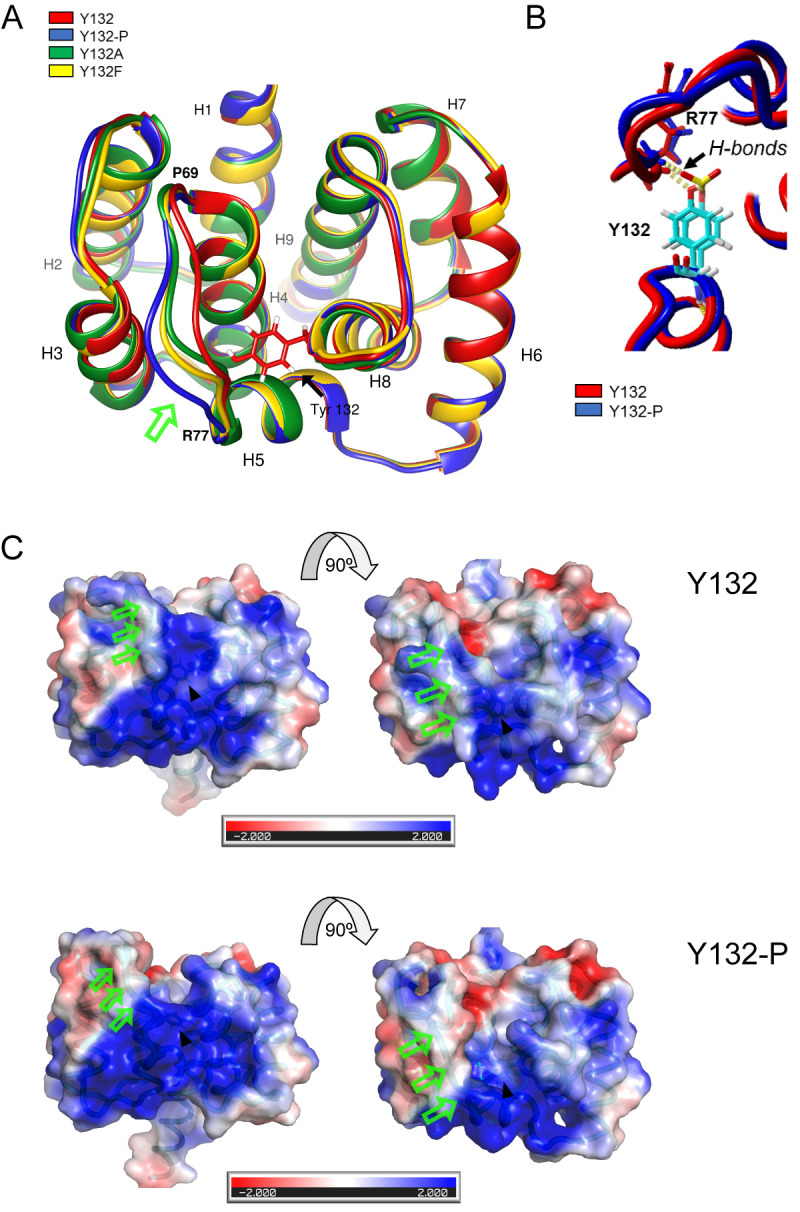
Phosphorylation of M1 Y132 induces a potential structural repositioning of the linker domain between helices 4 and 5. **A)** WSN M1 WT, pY132, Y132A and Y132F were modeled based on the solved and refined crystal structure (pdb: 5v8a) and all atoms mobile molecular dynamics simulations were performed. Average structures of WT and mutant models are colored as indicated and shown superimposed. **B)** Proposed hydrogen bond replacement resulting in a repositioning of the linker domain in M1 pY132. **C)** Surface representation of non-phosphorylated M1 in comparison to pY132, colored by the electrostatic potential calculated by Poisson-Bolzman with positive in blue and negative in red, viewed from different directions. Arrows highlight repositioned linker (green) and Y132 (black).

M1 functional transitions require partly profound conformational changes [2]. Time evolution of M1 carrying a phosphorylated Y132 (pY132, blue) was analyzed *in silico* using molecular dynamics simulations. The resultant structure was superimposed with non-phosphorylated M1 (red) on the basis of the published protein structure (PDB:5V8A) (Fig 2A). Non-phosphorylated Y132 is predicted to interact with 14 neighboring residues in a region of 5 Å present in helices 3, 4 and 5 (H3, H4 and H5). While the majority of these interactions seem to be conserved in the presence of pY132 (blue), the simulation suggests a new hydrogen bond formed between the third oxygen atom of the phosphate group (O3-pY132) and N-R77 (O-H distance ~2.1 Å) that replaces the hydrogen bond between OH-Y132 and N-R77 (O-H distance of 2.1 Å) (Fig 2B), resulting in a repositioning of the linker domain between H4 and H5, whereas the function of the aromatic group is stable (Fig 2A). Importantly, R77 is part of three consecutive basic arginine residues (R76-78) localized at H5 that have been described to be involved in the interaction with phospholipids at the inner surface of the plasma membrane [18,19]. Since this part of the linker domain is exposed on the surface, phosphorylation of Y132 might be a regulatory element to change linker position, thereby exposing or modifying interaction sites for other proteins, lipids or M1 oligomerization.

Electrostatic surface potential of M1 is relevant for interactions with membranes and M1 oligomerization. The responsible basic patch face of M1 monomers is formed by positively charged residues from H5, H6, H7 and H8 [18,20]. Analysis of the electrostatics of non-phosphorylated M1 (Fig 2C, top panels) in comparison to M1 pY132 (bottom panels) implies considerable modifications of the basic electrostatic surface potential, indicating that the phosphorylation of Y132 might affect M1 functions that are based on electrostatic attraction.

To exclude any aberrant structural changes due to the substitutions Y132A (green) or Y132F (yellow), these mutants were included in the simulation (Fig 2A). Overlay with non-phosphorylated and pY132 M1 showed no significant differences in tertiary structures, but highlighted a potential intermediate position of the linker domain. In summary, Y-to-A or Y-to-F substitutions do not seem to affect the intramolecular stability of M1. Thus, impaired replication of WSN M1 Y132A is not likely to be due to disturbed M1 protein folding induced by mutation.

### Presence of M1 Y132A induces the formation of filamentous particles

M1 shuttles between cytosol and nucleus in early stages of virus replication to modulate viral polymerase activity and guide newly synthesized vRNP complexes from the nucleus into the cytoplasm [6–8]. In late stages, M1 is mainly localized at the plasma membrane to initiate assembly and budding of progeny virions [3]. Due to the localization of the phospho-acceptor site Y132 at the surface of the M1 protein, a function of phosphorylation in all the different stages of viral replication would be conceivable (S2A Fig). While the data gained so far point to a late function of Y132 phosphorylation, Wang and colleagues previously observed in overexpression approaches a retention of the M1 Y132A substitution mutant in the cytoplasm due to diminished nuclear import of M1 that could be attributed to a reduced interaction with importin α [12]. In order to test whether an effect on early or intermediate M1 functions is also evident in genuine infection, subcellular localization of M1 was analyzed upon mutant virus infection. As expected, there were neither changes in M1 or vRNP localization between WSN M1 Y132A and WT infection at 5 or 7 hpi detectable (S2B and S2C Fig), nor was synthesis of different RNA species affected (S2D Fig). These data are consistent with viral protein levels (Fig 1E) and suggest that M1 Y132A amounts in the nucleus are sufficient to modulate viral polymerase activity to the same extent as WT M1. In addition, reduced viral replication in presence of M1 Y132A is not due to a disturbed switch of viral polymerase function from transcription to replication.

**Fig 3:**
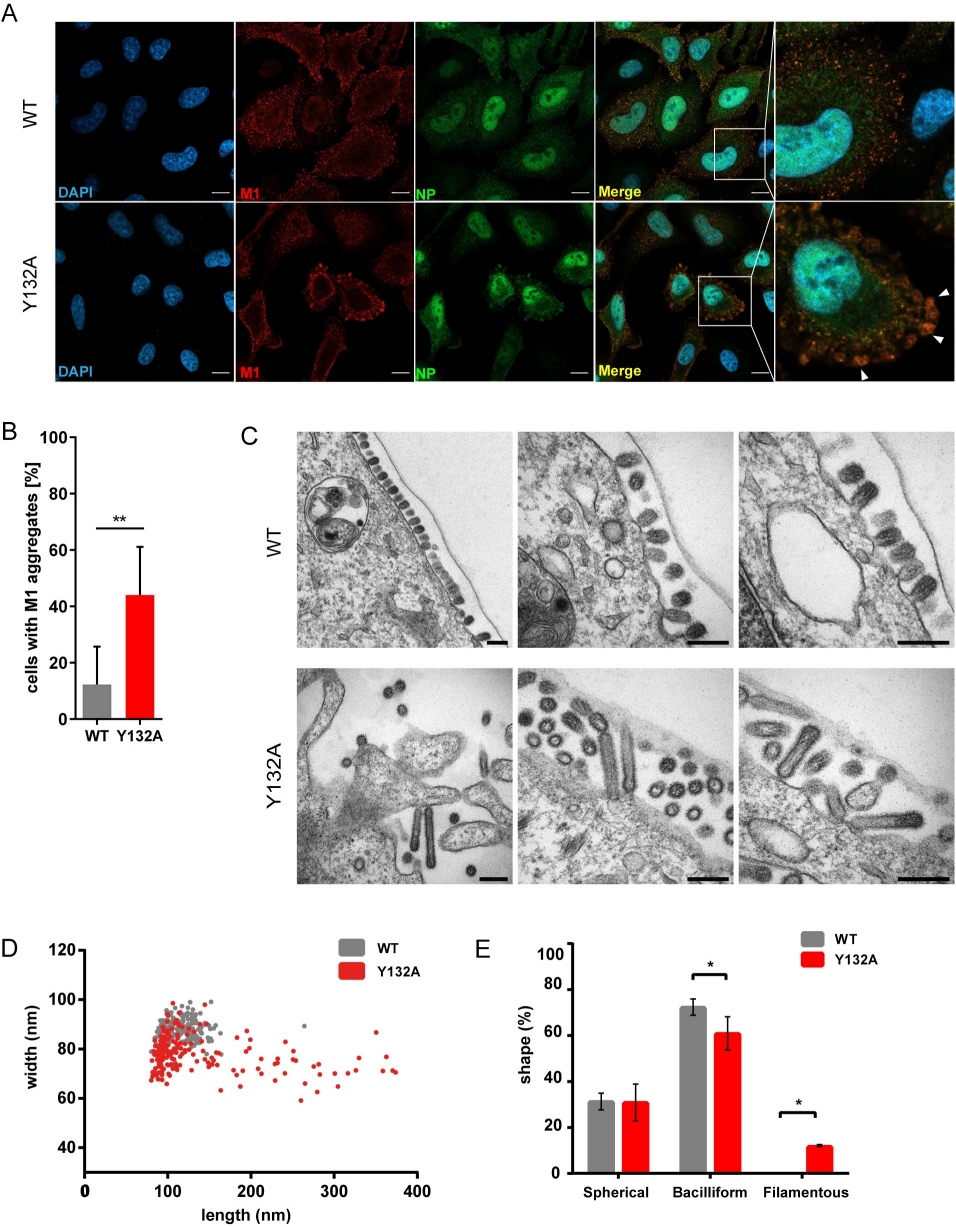
Presence of M1 Y132A induces the formation of filamentous particles. **A-E)** A549 cells were infected with WSN WT or M1 Y132A (MOI 5) for 9 h. **A)** Cells were fixed and analyzed for M1 localization (red) by indirect immunofluorescence. NP (green) was used as marker for vRNP localization and nuclei were stained with DAPI (blue). Pictures were taken with LSM800 confocal microscope and analyzed with Fiji/ImageJ version 1.51n. Scale bar: 10 μm. Arrows point to M1 aggregates at the cell periphery. Pictures are representatives of two independent experiments. **B)** Presence of M1 aggregates in WSN WT and M1 Y132A-infected A549 cells was quantified with Fiji/ImageJ version 1.51n. Results are depicted in percent of infected cells (n = 168 (WT) and n = 265 (mutant)). Significance was analyzed by using two-tailed unpaired t-test (**p≤0.01). **C)** Cells were fixed with 2.5% glutaraldehyde and proceeded for TEM. Samples were analyzed with a 120 kV FEI-Tecnai 12 TEM and pictures were analyzed by Fiji/ImageJ version 1.51n. Scale bar: 200 nm. **D)** Distribution of axial ratio of WSN WT (gray) and M1 Y132A (red) particles. Particles (n = 192) were randomly selected from 10 cells and measured in length and width. **E)** Morphology of WSN WT (grey) and M1 Y132A (red) virions. Particles were classified by axial ratio (length/width). Depicted are mean percentages ±SD of two independent measurements. Statistical analysis was performed by using two-way Anova followed by Sidak’s multiple comparisons test (*p<0.05).

At late stages of infection, WT M1 was localized in the cytosol near the plasma membrane (Fig 3A). Interestingly, M1 Y132A was also observed close to the membrane but accumulated in patches at the cell periphery, which was in part also true for NP. This phenomenon could also marginally be observed in WT virus infection (~13%), while it was detectable in ~45% of WSN M1 Y132A-infected cells (Fig 3B). These results suggest a potential arrest of M1 Y132A, which has been observed previously for R77/78A substitutions [18] localized in the presumably phosphorylation-induced repositioned linker. To further address the localization of M1 Y132A aggregates, the cellular membranous organelle hosting M1 patches was analyzed. However, neither the trans- or cis-Golgi network nor early endosomes could be identified to co-localize with M1 aggregates (S3 Fig). These results indicate that phosphorylation of Y132 does not alter M1 subcellular localization or function in early and intermediate stages of virus replication, but points towards an impact at late stages of virus life cycle progression.

Analysis of late events such as virion budding by transmission electron microscopy (TEM) revealed evident differences in the budozones of WT and mutant virus. Infection with WSN WT maintains a highly organized plasma membrane similar to mock-infected cells (S4A a, b, d Fig) and showed an ordered budding process. In contrast, pronounced membrane curvature with protrusions and stress fibers was particularly detected in WSN M1 Y132A infection (S4A c, e Fig). Strikingly, progression of budding events showed no evident impairment compared to WT (S4B Fig). However, TEM unraveled the presence of elongated particles in WSN M1 Y132A infection (Fig 3C), while the WT virus appeared in the usual spherical form reported for most laboratory-adapted strains. To quantify this phenotype, particles of both viruses were classified by axial ratio [21]. The respective plot illustrates a reduced width for most M1 Y132A-containing particles as well as an increase in length for some virions (Fig 3D). In presence of WT M1, 70% of particles presented a bacilliform shape (axial ratio 1.2–3.2) and 30% were spherical (axial ratio <1.2) (Fig 3E). In contrast, 11% of progeny virions produced by WSN M1 Y132A were classified as filamentous (axial ratio > 3.2), 30% were spherical and 59% bacilliform. Importantly, the axial ratio of these particles switched from bacilliform to filamentous, while the amount of spherical particles was not affected. Noteworthy, it was previously shown that more elongated particles can be related to an impairment or retardation in assembly/budding events [3,22]. Thus, the switch in viral particle morphology of the mutant is in line with a potential arrest of non-phosphorylated M1 at the cell periphery.

### Phosphorylation of M1 Y132 is required for lipid raft localization and increases structural stability of virus particles

**Fig 4:**
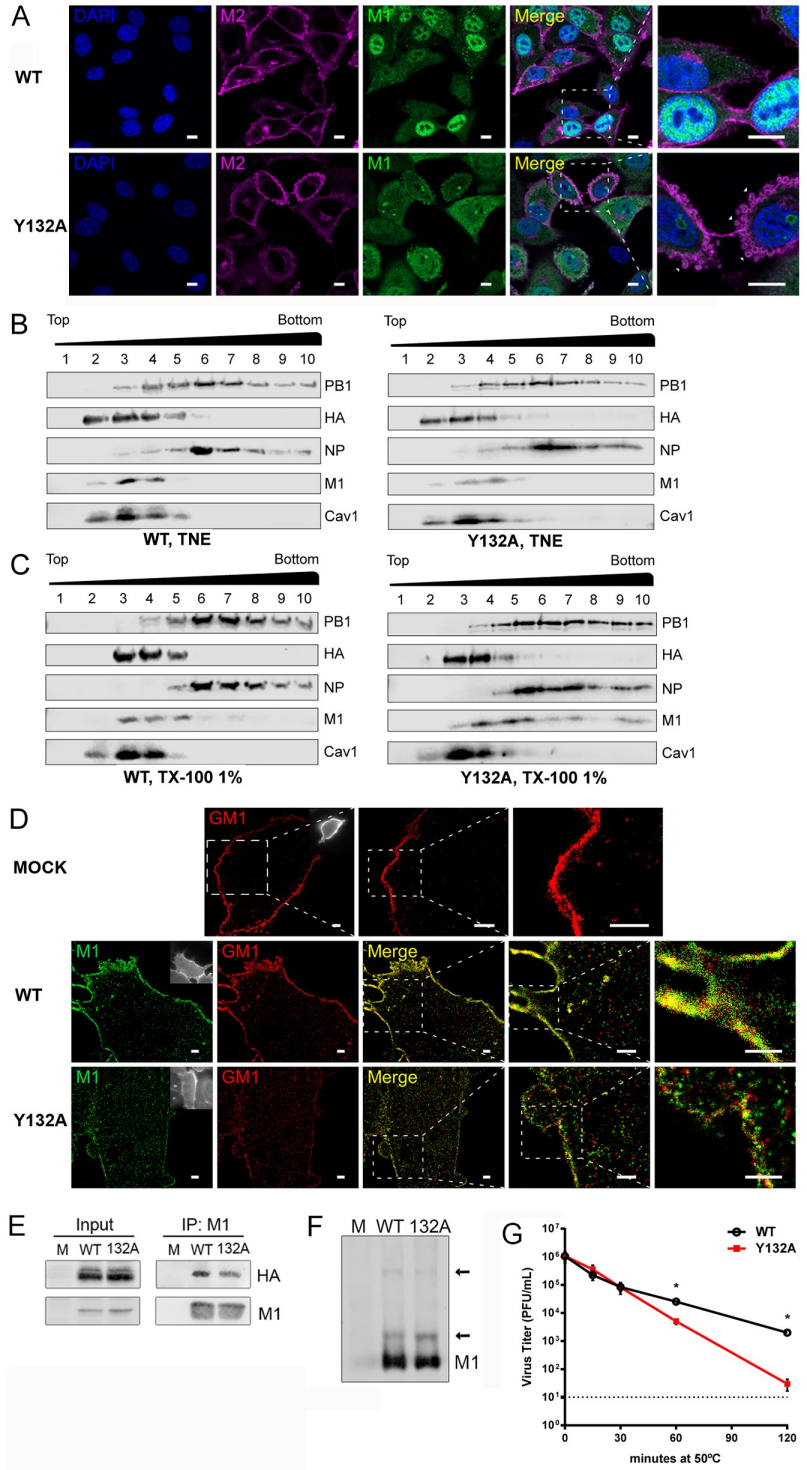
Phosphorylation of M1 Y132 is required for lipid raft localization and increases structural stability of virus particles. A549 cells were infected with 5 MOI WSN WT or M1 Y132A for 9 h **(A-E)**. **A)** Cells were fixed and analyzed for M1 (green) co-localization with M2 (purple) by indirect immunofluorescence. Nuclei were stained with DAPI (blue). Pictures were taken with LSM800 confocal microscope and analyzed with Fiji/ImageJ version 1.51n. Scale bar: 10 μm. Arrows point to M1 aggregates at the plasma membrane. Pictures are representatives of two independent experiments. **B)** Cells were lysed and subjected to membrane flotation assay. **C)** Lysates were resuspended in TNE buffer containing 1% (v/v) Triton-X100, incubated at 37°C for 30min and subjected to membrane flotation assay. **B, C)** Distribution of PB1, NP, HA and M1 was detected by western blot. Caveolin-1 was used as lipid raft marker. Representative blots of 5 independent experiments are shown. **D)** Cells were fixed and analyzed for M1 localization (green) by STORM. Mock-infected A549 cells served as control and GM1 (red) was used as lipid raft marker. One representative out of 10–11 randomly selected cells is shown. Scale bars: 3 μm, 1 μm and 0.5 μm. **E)** Cells were lysed and M1 was immunoprecipitated by using specific antibodies. Co-precipitation of HA was analyzed in western blot and M1 detection was used to confirm equal amounts of precipitated M1. Blots are representatives of three independent experiments. **F)** A549 cells were infected with 1 MOI of WSN WT or M1 Y132A. 16 hpi, cells were lysed and subjected to native PAGE. M1 detection in western blots was used to determine M1 polymerization. Arrows point to respective M1 polymerization bands. Blot is one representative out of three independent experiments. **G)** WSN WT and M1 Y132A viruses were diluted to approximately 1x10^6^ PFU/mL and incubated at 50ºC for the indicated periods of time. Titers were analyzed by standard plaque assays. Depicted are mean virus titers ±SD of three independent experiments with the dotted line indicating the detection limit. Statistical significance was determined by multiple *t* test for multiple comparison followed by Holm-Šídák correction (*p≤0.05).

M1 recruitment to the plasma membrane is needed to initiate virus assembly and budding [23]. Intracellular transport was shown to be mediated by binding of M1 to the cytoplasmic tail of viral M2 that carries M1 in a piggyback mechanism to the plasma membrane [24,25]. Here, M1 interacts with viral glycoproteins and the lipid bilayer leading to the recruitment of further M1 molecules and its oligomerization to form the matrix layer [2]. In order to investigate whether M1 Y132A is efficiently transported to the plasma membrane, we analyzed its co-localization with M2 (Fig 4A). Interestingly, M2 showed efficient membrane localization in infection with both, the WT and the substitution mutant, however, in cells with accumulating M1 Y132A, distinct membrane curvatures were observed that seem to imbed M1 Y132A patches. These results indicate that M1 Y132A efficiently reaches the membrane and confirm our previous observations made in TEM (S4A Fig) of a less organized budding process with a pronounced induction of membrane curvatures.

The next step in viral assembly and budding includes the interaction of M1 with the lipid bilayer. In this context, R76-78 have been shown to be crucial for M1 attachment to large unilamellar vesicles (LUVs) *in vitro* [19]. Coherently, we hypothesized that non-phosphorylated M1 at Y132 shows reduced affinity for lipid binding due to a potential lack of arginine triplet-containing linker repositioning (Fig 2A). To assess whether M1-lipid binding is affected by substitutions for Y132, interaction of recombinant WT M1 and Y132 phospho-site mutants (A, F or D) with LUVs was analyzed by co-sedimentation assays [19]. All three M1 mutants presented similar binding abilities to LUVs as WT M1 (S4C Fig), suggesting that the phosphorylation status of M1 Y132 does not modify M1-lipid binding.

In presence of IAV glycoproteins, M1 is mainly associated with lipid rafts in the plasma membrane [26]. Therefore, M1 localization to different cell membrane fractions in either WSN WT or WSN M1 Y132A infection was analyzed by membrane flotation assays. As marker for membrane fractions, cellular caveolin-1 was used, showing comparable localization as WT M1 and HA proteins, while viral PB1 and NP were mainly observed in the cytoplasmic fractions (Fig 4B, left panel). Interestingly, M1 Y132A was detected in the caveolin-1/HA fractions (Fig 4B, right panel), emphasizing its ability to efficiently associate with membranes. Total membrane fractions are a complex mixture of lipids and addition of detergent can separate soluble parts from insoluble fractions [27]. These detergent-resistant membranes (DRMs) are enriched in glycosphingolipids, sphingomyelin and cholesterol, together composing lipid raft microdomains, which are preferred sites of IAV assembly and budding [28]. Here, WT M1 was exclusively observed in insoluble fractions as revealed by caveolin-1 and HA (Fig 4C, left panel), which are both commonly used as specific markers for DRMs [14,29]. As expected, other viral proteins such as NP and PB1 showed a slight shift to more soluble fractions when compared to non-detergent conditions. Strikingly, M1 Y132A was not exclusively observed in DRMs but also in soluble fractions (Fig 4C, right panel), while all other viral proteins tested were observed in the same fractions as in WT infection. These data suggest that non-phosphorylated M1 Y132 maintains the ability to associate with lipids and cell membranes, nevertheless, phosphorylation is required for efficient lipid raft localization.

To take a closer look on M1 localization at the plasma membrane, super-resolution microscopy was performed. As marker for lipid raft domains, sphingolipid GM1 detection was used (Fig 4D) [30]. Here, GM1 was mainly observed in the plasma membrane in non-infected (upper panel) as well as in WSN WT-infected cells (middle panel) and M1 Y132A-infected cells. In contrast to WT, M1 Y132A showed a significantly reduced degree of co-localization with GM1 at the plasma membrane. With higher resolution, differences in the distribution of single M1 molecules can be observed that might correspond to M1 Y132A that accumulated at the plasma membrane, as observed previously by confocal microscopy (Fig 3A). After reaching the plasma membrane, M1 is recruited to viral assembly sites by interaction with glycoproteins HA and NA [26,31]. Indeed, in M1 Y132A infection, decreased amounts of HA were co-precipitated with M1 (Fig 4E), suggesting that reduced lipid raft localization of M1 Y132A can be attributed to a diminished recruitment via HA.

Association of M1 with plasma membranes triggers its oligomerization as a consequence of an increased affinity to other monomers [32]. Therefore, M1 polymerization was analyzed under non-denaturing conditions, indicating a comparable dimer- and polymerization as observed in WT virus infection (Fig 4F). However, the matrix layer preserves the integrity of virus particles and supports the fragile lipid envelope [2]. Thus, changes in M1 localization or the interaction with other viral proteins can affect structural stability of virus particles. To evaluate matrix layer integrity, heat resistance was assessed by exposing the same amount of infectious particles of mutant and WT virus to 50ºC followed by the analysis of the remaining infectivity. Infectivity of M1 Y132A-containing particles considerably decreased after 60 min with viral titers declining to the detection limit after 120 min, where infectious WT viruses were still detectable (Fig 4G). Consequently, WSN particles with non-phosphorylated M1 Y132 manifest a less stable structure, which might indicate aberrant matrix layer formation induced by an impaired M1-HA interaction and a diminished M1 localization at the budozone.

### Proposed M1 Y132 phosphorylation-induced repositioning of the linker region enlarges a positively charged groove in M1 dimers

**Fig 5:**
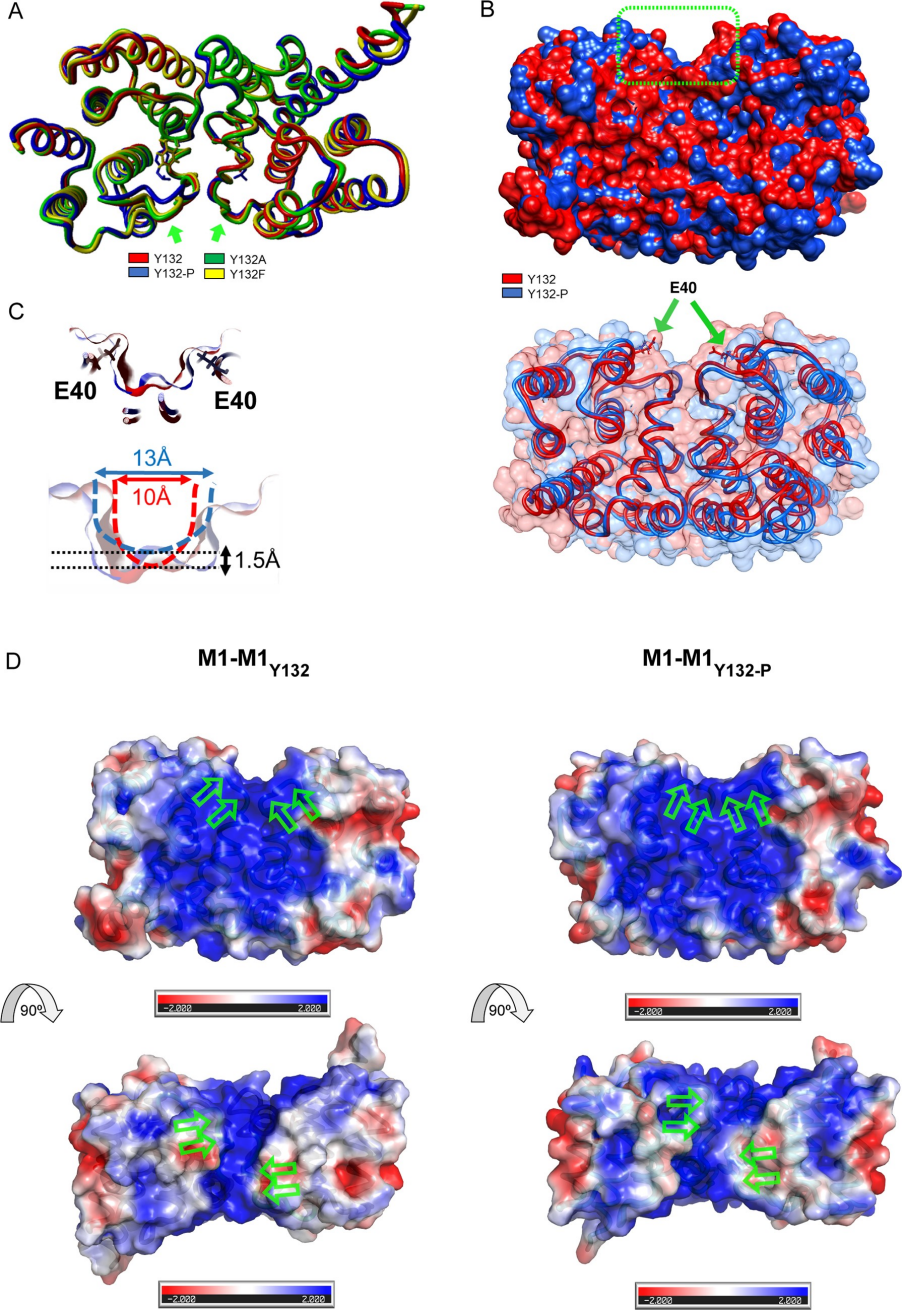
Proposed M1 Y132 phosphorylation-induced repositioning of the linker regions enlarges a positively charged groove in M1 dimer interfaces. **A)** Homodimers of WSN M1 WT, pY132, Y132A, and Y132F were modeled based on the solved and refined crystal structure (pdb: 5CQE) and all atoms mobile molecular dynamics simulations were performed. Average structures of WT and mutant models are colored as indicated and shown superimposed. **B)** Surface representation of M1 homodimers revealing the potential presence of a central groove at the interface of M1 monomers with E40 located at the center. **C)** Changes in groove width and depth induced by pY132. **D)** Surface representation of non-phosphorylated (left) and pY132 (right) homodimeric M1, viewed from two different directions. Molecules are colored by electrostatic potential calculated by Poisson-Bolzman with positive in blue and negative in red. Arrows highlight repositioned linkers.

M1 energetically accesses different mono- and dimer conformations, as well as oligomeric states, with varying degree of similarities. Generally, there are two major subpopulations of M1 dimers described: (I) dimers composed of NM-domains stacked in face-to-back fashion and (II) M1 monomers oriented in a face-to-face dimeric structure [33,34]. In the face-to-back orientation, electrostatic interactions between the basic NLS region localized in H6 and nearby basic residues are maximized with a cluster of negatively-charged residues of the opposite M1 face [33]. In contrast, the face-to-face orientation allows for hydrophobic interactions between the respective H6 of M1 monomers [34]. In the face-to-back subpopulation, dimer formation should not be significantly modified by Y132 phosphorylation, as the linker between H4 and H5 is not part or in close proximity of the shared interfacial surface area. Time evolution of phosphorylation-induced changes in face-to-face dimers was analyzed in molecular dynamics simulations (PDB: 5CQE) (Fig 5A). Comparison between non-phosphorylated and pY132 M1 clearly showed the same repositioning of the linker domain between H4 and H5 as observed in monomeric M1. Interestingly, the function of the aromatic group was no longer stable, but eluded from the interaction site. Comparison with the substitution mutants Y132A and F did not show an intermediate phenotype for linker repositioning as observed previously, but fully coincided with non-phosphorylated M1. Further analysis deciphered the potential existence of a central groove at the interface of M1 monomers with aa E40 located at the center (Fig 5B). In the non-phosphorylated situation, this groove seems to be narrow and steep, while pY132-induced linker repositioning might lead to an opening, resulting in a wider, shallower groove (Fig 5C). Interestingly, this groove comprises a net positive charge that could be more accessible upon Y132 phosphorylation (Fig 5D). Consequently, phosphorylation of M1 Y132 does not seem to modify efficient dimer formation as already observed in non-denaturing PAGE, but might be involved in the exposition of a positively charged potential interaction site in M1 dimers that might facilitate electrostatic interactions to orchestrate viral budozones. Furthermore, this positively charged interaction site would qualify for the recruitment of (negatively charged) viral genomic RNA (vRNA) during the process of virus assembly.

### Phosphorylation of M1 Y132 is required for efficient vRNP incorporation into budding virions

**Fig 6:**
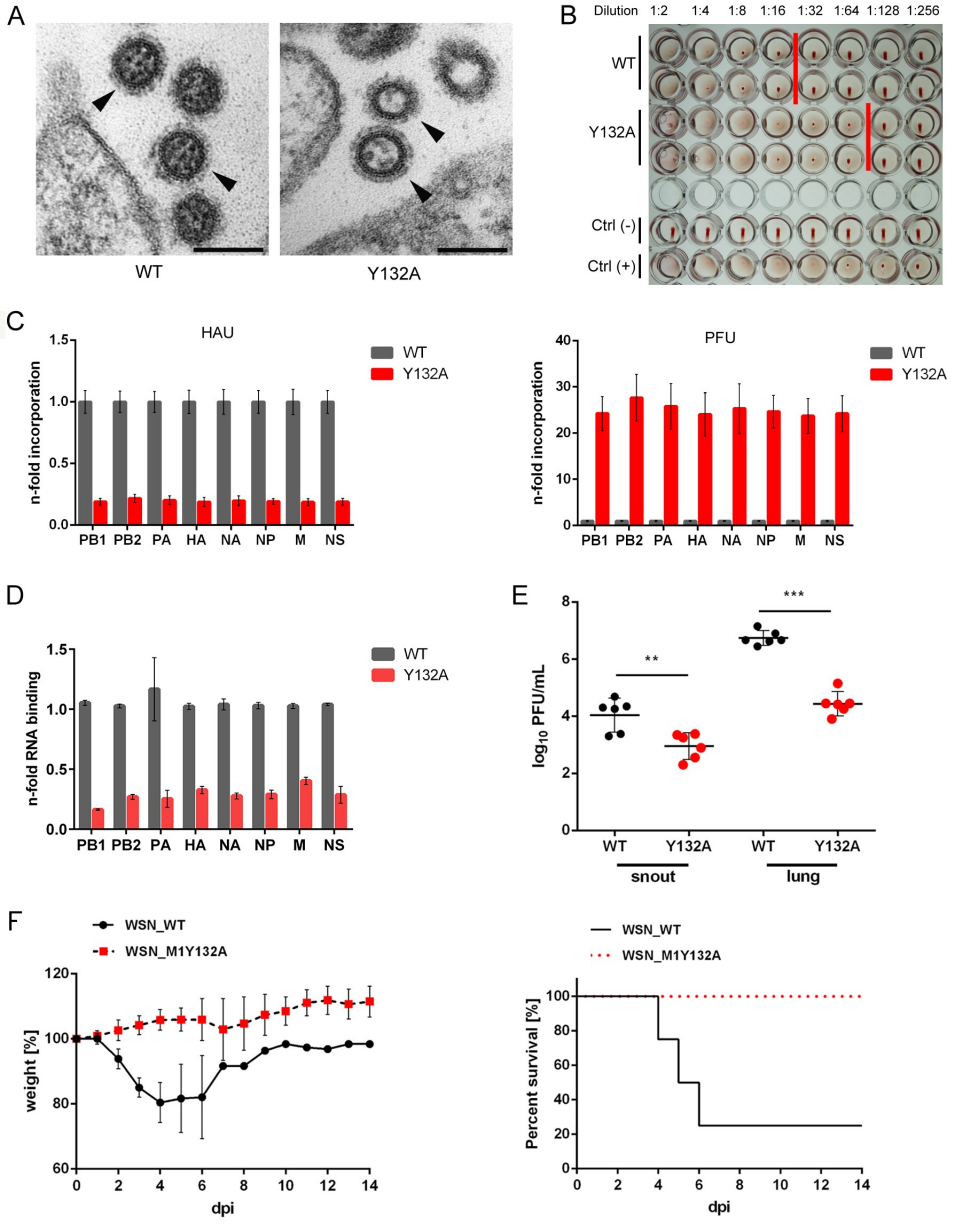
Phosphorylation of M1 Y132 is required for efficient vRNP packaging into budding virions and conveys virus pathogenicity in mice. **A)** A549 cells were infected with WSN WT or M1 Y132A (MOI 5) for 9 h. Cells were fixed with 2.5% glutaraldehyde and processed for EM as described for Fig 3. Scale bar: 100 nm. **B)** Analysis of the relative amount of viral particles in supernatants from WSN WT or M1 Y132A-infected MDCKII cells by HA assays with a defined number of 7x10^5^ infectious particles. One representative of two independent experiments is shown. **C)** Virus-containing supernatants of WSN WT- or M1 Y132A-infected MDCKII cells (0.1 MOI; 48 hpi) were harvested, infectious titers were determined by standard plaque assays and HAU were analyzed by HA assays. Incorporation of vRNAs into viral particles was analyzed by qRT-PCR and normalized to the total amount of particles (HAU/pfu; left) or the amount of infectious particles (right). vRNA levels of WSN WT particles were arbitrarily set to 1. Depicted are mean vRNA incorporation levels ±SD of three independent experiments. **D)** A549 cells were infected with 5 MOI of WSN WT or M1 Y132A and lysed 9 hpi. M1 was immunoprecipitated and amounts of bound viral RNA were analyzed by qRT-PCR after TriFast isolation of RNA. vRNA binding capacity of WT M1 was arbitrarily set to 1. Depicted are mean vRNA levels ±SD of three independent experiments. **E, F)** hMxA-tg mice were inoculated intranasally with WSN WT or M1 Y132A (5x10^5^ pfu in 40 μl). **E)** Viral lung titers were determined 3 dpi. Individual animals are depicted. Mann-Whitney test (**p≤0.01, ***p≤0.001). **F)** Body weight curves (left); mean % body weight of 1–4 (initial group size) animals normalized to initial weight +/-SD is depicted. Animals were excluded from the analysis when reaching less than 75% of initial body weight. Survival curves (right); %-survival of 1–4 (initial group size) animals is shown.

Virus assembly does not only include efficient recruitment of viral proteins to the respective budozones, but also involves packaging of viral genomes. Strikingly, selective incorporation of vRNPs in the virion is supported by the matrix scaffold [35]. vRNPs incorporated in released particles present a “7+1” configuration, as seven segments are surrounding an eighth in the center [36]. These characteristic vRNP arrangements were observed in TEM of WSN WT particles (Fig 6A). In contrast, some virions from WSN M1 Y132A infection were lacking regular vRNP configuration or even seemed to be empty. To understand whether these observations are based on a defect in genome incorporation or only due to sample sectioning in TEM, the generation of non-infectious progeny of WT and mutant virus was compared by hemagglutination (HA) assay. Here, an increased amount of virus particles was observed, which was elevated by a factor of 4 for WSN M1 Y132A infection compared to WT (Fig 6B). To address the efficiency of segment incorporation, vRNA levels for each individual segment were quantified from released virus particles by qRT-PCR. vRNA incorporation was normalized to the total number of particles produced as determined by HA assay. Here, WSN M1 Y132A showed diminished incorporation for all eight segments compared to WT (Fig 6C). Interestingly, vRNA levels normalized to the amount of infectious particles were approximately 25 times higher in the presence of M1 Y132A. These results highlight a random segment incorporation defect of budding virions, resulting in the production of non-infectious particles. To minimize the levels of defective particles produced due to the von Magnus effect [37], viruses analyzed for vRNA incorporation were propagated using a low MOI of 0.001. Surprisingly, defects in genome incorporation were even more pronounced at lower MOI (S4 Fig). Thus, generation of non-infectious particles in presence of M1 Y132A is not based on error-prone viral polymerase activities, but rather phosphorylation of M1 Y132 is needed for efficient genome incorporation into budding virions. Indeed, M1 Y132A showed a significantly reduced binding capacity for viral RNA (Fig 6D), indicating that the potential exposition of the positively charged linker region might increase the binding affinity of M1 for viral genomic segments which is needed for efficient genome incorporation into viral progeny.

### M1 Y132 phosphorylation conveys virus pathogenicity in mice

To analyze whether phosphorylation of Y132 affects disease severity of WSN infection, human MxA transgenic mice (hMxA-tg) were infected with WT or M1 Y132A-expressing viruses. These mice lack functional endogenous *Mx* genes but instead carry the human *MX1* locus as a transgene, thereby better reflecting the natural situation since these mice exhibit moderate resistance to IAV of human origin [38]. Infection of these mice with WSN M1 Y132A led to significantly decreased virus titers compared to WT in both, snout and lung at 3 dpi (Fig 6E). To analyze whether this decrease in viral replication is sufficient to protect mice from lethal infection, body weight loss and survival were determined. While in the presence of WT, mice lost body weight as early as 2 dpi, weight loss in WSN M1 Y132A-infected mice was not detectable (Fig 6F, left panel). This is supported by the survival curves, which show survival of all mice infected with WSN M1 Y132A, whereas 75% mortality was observed in WT-infected mice (Fig 6F, right panel). These results clearly demonstrate that the defects observed in viral replication in absence of M1 Y132 phosphorylation translate to a strongly reduced viral pathogenicity in mice.

## Discussion

In this study, we focused on tyrosine phosphorylation as dynamic regulatory mechanism of IAV M1 structural diversification and multifunctionality, which was evaluated by creating recombinant viruses carrying non-phosphorylatable aa at position Y10 or Y132. Both tyrosines are exposed on the surface and are prone to phosphorylation at late stages of virus replication. While viruses carrying M1 Y132A presented significantly reduced viral replication compared to WT *in vitro* and *in vivo*, M1 Y10 phosphorylation seems to be dispensable. By detailed analysis, we uncovered that phosphorylation of M1 Y132 seems to be mediated by Janus kinases. JAKs are predominantly located at membranes [39] and have previously been shown to be needed for efficient viral replication [16,17]. The present study reveals a late phosphorylation of M1 Y132 during viral life cycle progression, most likely occurring when M1 reaches the plasma membrane. This regulatory mechanism illustrates the high degree of adaptation of enveloped viruses to their mammalian hosts by using a membrane-associated cellular kinase that initiates virus assembly by phosphorylation of viral matrix protein, the major driving force of virus budding.

Proper virus assembly and budding of progeny virions is achieved by finely regulated complex interactions of several viral proteins, and M1 appears to orchestrate the spatial and temporal order of these events [3]. Here, prevention of M1 Y132 phosphorylation led to protein aggregation at the plasma membrane in late stages of infection. This accumulation in patches at the cell periphery has been reported previously for mutations of aa R76-78 [18,19]. This triplet belongs to the patch of residues with positive charge, which was shown to be directly involved in M1 association with membranes and in M1-M1 interactions [20,40,41]. Here, substitution for Y132 did not change lipid binding, but M1 Y132A was observed in non-DRM fractions. Interestingly, phosphatidylinositol 4,5-bisphosphate has been shown to be enriched in membrane regions with DRM-resident viral HA protein [42]. It may be hypothesized that these negatively charged lipids pose a docking site for M1 pY132 after exposure of the positively charged linker region.

Upon association with the plasma membrane, M1-M1 interactions are facilitated, leading to oligomerization and matrix layer formation. M1 polymerization can form lattices within a range of curvatures influencing the final morphology of progeny virions [43]. In addition, the M1 protein layer is the most rigid structure of the virion and, therefore, determines structural stability of virus particles. Specifically, basic residues of the linker loop and the M-domain interact with negatively-charged residues from the N-terminal domain of the other monomer [44]. Apparently, strongest interactions in the matrix layer occur between regions of the NM- and C-terminal domains of one monomer with the NM-domains of a second monomer [34], facilitated by supportive interactions between the NM-domains of neighboring monomers. This indicates that polymerization is a dynamic interaction between the NM- and C-terminal domains of M1 monomers. Taking into account that Y132 is present in the M-domain and that its phosphorylation modifies virion structure as well as structural stability of the matrix layer, a role of M1 Y132 phosphorylation in the exposure of the positively charged face of M1 monomers to facilitate M1-M1 interactions and the formation of the matrix layer is highly conceivable. Nevertheless, we did not observe diminished M1 polymerization for non-phosphorylatable M1 Y132A, but rather the tendency of accumulation that might be attributed to its diminished recruitment to lipid raft domains via the viral HA protein. M1 is known to be localized in lipid rafts in a HA or NA dependent manner [26]. Since M1 lacks an inherent membrane targeting signal [31], it is thought to be co-transported to the cell surface with other viral components. Thus, an impaired interaction with HA in the presence of non-phosphorylated M1 Y132 seems to affect M1 localization in DRMs. So far, no exact aa residues responsible for these interactions were reported, nevertheless, it is highly conceivable that PTMs of M1 influence its binding ability to viral glycoproteins, which has been proposed to possibly already occur at intracellular membranes [45,46]. Interestingly, we observed efficient transport to the membrane, most likely in a M2 dependent manner, supporting the hypothesis of M2-mediated intracellular transport in a piggyback mechanism [24,25]. Here, presence of M1 Y132A led to pronounced changes in membrane curvature, and M1 seemed to accumulate in these membrane bendings. It should be mentioned that, so far, we cannot fully exclude an influence of the introduced substitution of alanine for tyrosine rather than the missing phosphorylation at Y132 alone.

The process of viral genome incorporation into budding virions is still not completely understood, but current evidence supports an involvement of M1 [35,47]. Interestingly, efficient vRNP recruitment to viral budozones indeed appears to be dependent on phosphorylation of M1 Y132 as all the eight viral segments were less efficiently incorporated into progeny particles in the presence of M1 Y132A. This can be attributed to a reduced RNA binding affinity in absence of phosphorylation, resulting in a general genome incorporation defect. Substantial evidence showed that the M- and C-domains of M1 are involved in the binding of vRNPs [41,44] and the M-domain was shown to possess an intrinsic RNA-binding property [48,49]. So, molecular dynamic simulation proposed a mechanism by which phosphorylation-induced surface exposition of the positively charged linker region might be involved in efficient vRNP incorporation.

This work represents one of the few studies characterizing posttranslational modifications of IAV M1 by generation of recombinant viruses carrying mutant phenotypes and highlights the importance to functionally characterize modifications of viral proteins for a detailed understanding of viral replication. Here, we provide evidence that M1 Y132 phosphorylation is involved in the regulation of assembly and budding events. Thus, targeting JAKs as the presumably responsible kinases might be a promising approach for antiviral intervention that might ensure interference with replication of a broad range of influenza viruses with a high barrier towards generation of resistant variants.

## Methods

### Cell lines

All cell lines used in this study were originally purchased from ATCC and have been passaged in the laboratory. At regular intervals cells are checked for their identity by SNP-profiling (Multiplexion). Human embryonic kidney 293 (HEK293T) and human alveolar lung epithelial (A549) cell lines were grown in Dulbecco’s modified Eagle medium (DMEM). Madin-Darby canine kidney epithelial (MDCKII) cell line was grown in Minimum Essential medium (MEM). All media were supplemented with 10% fetal bovine serum (FBS) and cell lines were incubated at 37°C in 5% CO_2_ atmosphere. HEK293 and MDCKII cell lines are of female origin, while the A549 cell line is of male origin.

### Ethics statement

Female and male hMx-tg mice used in this study were bred in the facility of the Institute of Virology Freiburg with 12 h light/dark cycle and continuous access to food and water. This transgenic mouse carries a bacterial artificial chromosome that includes the entire human *MX* locus and founder animals were backcrossed to C57BL/6 mice lacking functional endogenous *Mx* genes [38]. All animal experiments were performed in accordance with the guidelines of the German animal protection law and were approved by the state of Baden-Württemberg (Regierungspräsidium Freiburg; reference number: G12/46). Littermates of the same sex were randomly assigned to experimental groups. Infections of 8 to 10 weeks old hMx-tg mice were performed under BSL-2 conditions in line with the local animal care committees by using a mixture of ketamine (100 mg per g body weight) and xylazine (5 mg per g body weight) administered intraperitoneally. Throughout the course of the experiment, mice were monitored daily and if severe illness or weight loss of >25% occurred, mice were sacrificed by cervical dislocation.

### Virus infection

HEK293T, MDCKII and A549 cells were infected with WSN wild type or mutant viruses in PBS for infection (1% Penicillin/Streptomycin, 0.2% (v/v) Bovine Serum Albumin (BSA 35%), 0.01% MgCl_2_, 0.01% CaCl_2_) at the indicated MOIs of 0.01–5 for 30 minutes at 37ºC. Virus inoculum was removed and monolayers were washed twice with PBS. DMEM or MEM medium supplemented with 1% Penicillin/Streptomycin, 0.2% (v/v) Bovine Serum Albumin (BSA 35%), 0.01% MgCl_2_, 0.01% CaCl_2_ and 0.15 μg TPCK-treated trypsin was added and plates were incubated at 37ºC. Supernatants or cell lysates were harvested at the indicated time points. For immunofluorescence, electron microscopy and membrane flotation assays, synchronization of infection was performed at 4ºC for 30 min. Afterward, virus inoculum was removed and cells were washed with PBS. Subsequently, PBS for infection was added to allow the attached virus to enter the cells for 30 min at 37ºC.

### Generation of recombinant viruses

WSN M1 Y132A and Y10F were generated by using a set of eight plasmids based on the bidirectional pHW2000 plasmid reverse genetics system. Vectors carrying the amino acid changes from tyrosine (TAC) to alanine (GCC) in position 132 and from tyrosine (TAC) to phenylalanine (TTC) in position 10 were generated by site-directed mutagenesis. Primer sequences are listed in S1 Table. Confluent co-cultures of HEK293 and MDCKII cells were transfected with 1μg DNA of each plasmid encoding the eight viral segments by using Opti-MEM and Lipofectamine 2000. Medium was removed 16 h post-transfection and fresh DMEM supplemented with 1% Penicillin/Streptomycin, 0.2% (v/v) Bovine Serum Albumin (BSA 35%), 0.01% MgCl_2_, 0.01% CaCl_2_ and 0.15 μg TPCK-treated trypsin was added. Monolayers were analyzed every 24 h for cytopathic effects induced by efficient virus replication. Supernatants were harvested, centrifuged for 10 min at 4000 rpm, 4°C and titrated by standard plaque assays. Viruses were plaque-purified and further propagated on MDCK monolayers.

### Virus titration by standard plaque assay

MDCKII were grown in 6-well plates till full confluency. Serial 10-fold dilutions of virus-containing supernatants were used to infect MDCKII and incubated for 30 min at 37°C. Virus inoculum was then removed and replaced by plaque medium (14.2% (v/v) 10× MEM, 0.3% (v/v) NaHCO_3_ (7.5%), 0.014% (v/v) DEAE-dextran (1%), 1.4% (v/v) 100× Penicillin/Streptomycin, 0.3% (v/v) BSA (35%), 0.9% (v/v) Agar (3%), 0.01% (v/v) MgCl_2_ (1%), 0.01% (v/v) CaCl_2_ (1%), 0.15 μg TPCK-Trypsin). Cells were incubated upside-down for 72 h at 37°C.

### Hemagglutination assay

Virus-containing cell supernatants were used to quantify the amount of total particles produced upon infection with WSN WT or M1 Y132A. HA assays were performed in V-bottomed microtiter plates. When needed, the same amount of infectious particles (PFU) was used and adjusted to a final volume of 50 μl with PBS. Virus samples were serially diluted (1:2) in PBS until dilution 1:1024 and 50 μl of fresh 1% chicken erythrocytes in PBS were added to each well. Plates were incubated at 4ºC for 1.5 h. The reciprocal of the highest dilution showing efficient agglutination of erythrocytes is reported as HA titer (hemagglutinating unit, HAU). WSN WT particles in high concentrations (1.0x10^8^ PFU/mL) and PBS were used as positive and negative controls, respectively. Hemagglutination was monitored by photography.

### Western blot analysis

Infected or transfected cells were washed twice with PBS and lysed in radioimmunoprecipitation assay (RIPA) buffer (25 mM Tris-HCl [pH 8], 137 mM NaCl, 10% Glycerol, 0.1% SDS, 0.5% Sodium deoxycholate, 1% NP-40, 2 mM EDTA [pH 8]) supplemented with protease inhibitor cocktail. The lysates were cleared by centrifugation at 20,000 g for 15 min at 4ºC. Relative protein concentration was measured by Bradford protein assay and 5x Laemmli buffer was added. Equal amounts of total protein were separated by SDS-PAGE and transferred onto nitrocellulose membranes. 5% BSA diluted in TBS-T buffer (50 mM Tris-HCl [pH 7.5], 150 mM NaCl, 0.2% Triton X-100) was used for blocking. Primary and secondary antibodies were diluted in TBS-T buffer supplemented with BSA (3% w/v), added to the membranes and incubated overnight or one hour, respectively. Documentation using fluorescence or chemiluminescence was performed with Li-Cor Odissey Fc Imaging System and semi-quantification of protein expression was performed using the software Image Studio.

### siRNA-mediated gene silencing

Human *JAK1* siRNA (5′-GCCUGAGAGUGGAGGUAA-3′), human *JAK2* siRNA (5′-GAACAGGAUUUACAGUUA-3′) as well as control siRNA (5′-UUCUCCGAACGUGUCACGU-3′) were synthesized by Eurofins Genomics GmbH. Transfection of A549 cells was performed with Lipofectamine 2000 (Invitrogen) according to the manufacturer's protocols.

### Generation of recombinant M1 protein

pGEX-6P-1 plasmids carrying the ORF of WSN M1 WT or M1 Y132 mutants (A, F, D) were transformed into E. coli BL21 and cultured overnight in 100 ml Luria broth supplemented with ampicillin (100 mg/ml) at 37ºC. 5 ml of cultures were transferred to fresh medium supplemented with 500 μl ampicillin and incubated at 37ºC. When an optical density of 0.6 was reached, 0.1 mM Isopropyl β-D-1-thiogalactopyranoside (IPTG) was added to stimulate protein expression and bacteria were incubated at 25ºC with shaking at 100 rpm for another 20 h. 5 ml of Glutathione Sepharose 4B beads were washed twice with water and 2x with resuspension buffer (50 mM Tris-HCl [pH 7.5], 100 mM NaCl, 1 mM EDTA), and kept at 4ºC until use. After incubation, bacterial cultures were cooled down and pelleted by centrifugation at 4000 rpm for 30 min at 4ºC. Pellets were resuspended in 20 ml resuspension buffer supplemented with protease inhibitor cocktail. Bacteria were lysed by 3x ultra sonication for 30 s. Lysates were supplemented with 1.25 ml 20% Triton X-100 and 10 mM β-mercaptoethanol and incubated at constant rotation for 15 min at 4ºC to ensure complete lysis. Lysates were cleared by centrifugation at 15000 rpm (SS34 Sorvall) for 30 min at 4ºC. Cleared lysates were supplemented with protease inhibitor cocktail and Glutathione Sepharose 4B beads were added. Interaction was allowed by rotation for 2 h at 4ºC. After this, beads were pelleted at 2600 rpm (Eppendorf 5417R) for 3 min at 4ºC. Beads were washed 6x with washing buffer (50 mM Tris-HCl [pH 7.5], 100 mM NaCl, 10 mM β-ME, 1 mM EDTA, 0.1% (v/v) Tween 20) and pelleted at 2600 rpm for 3 min at 4ºC after each washing step. Proteins were eluted using elution buffer (50 mM Tris-HCl [pH 8.0], 20 mM Glutathione) and protein concentration was measured spectrophotometrically using NanoDrop. When required, protein buffer was exchanged to TRIS-buffer (25 mM Tris [pH 7.5], 200 mM NaCl, 0.079%, glycerol, 5 mM β-mercaptoethanol) by dialysis.

### *In vitro* kinase assay

0.5 μg of recombinant JAK2 kinase domain were incubated in 30 μl kinase buffer [20 mM Tris/HCl (pH 7.5), 50 mM MgCl_2_, 1 mM DTT, 0.1 mM ATP] together with 2 μg of recombinant M1 for 30 min at 30°C. The reaction was stopped by addition of 5 × Laemmli buffer and denaturation of the proteins at 95°C for 5 min. Thereafter, phosphorylation and presence of proteins was analyzed by SDS-PAGE and Western Blot.

### M1 co-sedimentation assays with large unilamellar vesicles

Binding of M1 WT and Y132 mutants (A, F and D) to negatively charged lipids was determined by co-sedimentation assays using large unilamellar vesicles (LUVs). LUVs were generated with POPC (1-palmitoyl-2-oleyl-sn-glycero-3-phosphocholine), DOPS (1,2-dioleyl-sn-glycero-3-phospho-L-serine) and Cholesterol. DOPS, POPC, and cholesterol at a molar ratio of 40:40:20 were dissolved in 5 ml methylene chloride in a round-bottomed flask at a total lipid concentration of 10 mM. Methylene chloride was evaporated for 16 min at 60°C and 800 mbar. The lipid layer was stored overnight in a drying chamber at 40°C to evaporate residual organic solvent. 5 ml TRIS-buffer (25 mM Tris [pH 7.5], 200 mM NaCl, 0.079% (v/v) glycerol, 5 mM β-mercaptoethanol) and glass beads were used for rehydration of the lipid layer in the flask. LUVs were prepared by lipid film hydration followed by membrane extrusion and were characterized by a diameter of 178.6 ± 2.0 nm and a PDI of 0.21 ± 0.02 using the 0.22 μm filter and 233.6 nm (PDI of 0.38) using the 0.45 μm filter, respectively.

Recombinant M1 wildtype or the different mutant proteins were incubated with LUVs in a proportion of 1:150 in KCl/Hepes buffer (150 mM KCl, 0.5 mM EDTA, 20 mM Hepes [pH 7.4]) in a final volume of 100 μl at room temperature for 1 hour. Samples were centrifuged at 16ºC for 20 min at 70,000 rpm (TLA 100.2, Beckman Coulter). Samples were divided in supernatants (90 μl) containing unbound M1, and the pellet (10 μl) containing M1 bound to LUVs. Pellet fractions were diluted in 80 μL of KCl/Hepes buffer to maintain the equivalence between both fraction volumes. 5x Laemmli buffer was added, cooked at 95ºC for 3 min and resolved by SDS-PAGE and stained with Coomassie Blue. Intensity (I) of M1 bands from supernatant (S) or pellet (P) fractions was quantified by densitometry using Image Studio. The percentage of LUV-bound M1 was calculated as: % M1 LUV bound = 100*I_P_/(I_P_+I_S_).

### Membrane flotation assay

A549 cells grown on 6-cm dishes were infected with WSN WT or mutant virus at a MOI of 5 for 9 h and, subsequently, cells were scraped and resuspended in cold PBS. Cells were pelleted for 2 min at 2000 rpm and resuspended in 300 μl TE Buffer (10 mM Tris-HCl [pH 7.5], 4 mM EDTA and protease inhibitor cocktail). The cells were lysed by sonication in a pre-chilled ultrasonic processor (UP200St) by 3 series of 20 strokes, 0.5 s per stroke at maximal amplitude. The post-nuclear supernatant (PNS) was obtained by centrifugation for 3 min at 2000 rpm at 4ºC. Relative protein concentration was measured by Bradford protein assay and PNS was adjusted with TE buffer to an equal amount of total protein for each sample. Subsequently, samples were split into three aliquots of 90 μl each. Salt concentration was adjusted to 150 mM NaCl and the aliquots were mixed with 90 μl of TNE (25 mM Tris-HCl [pH 7.5], 150 mM NaCl, 4 mM EDTA and protease inhibitors) or TNE-T (TNE with 0.5% (v/v) Triton X-100), and the mixtures were incubated for 30 min at 0ºC or 37ºC, respectively. Afterward, 160 μl of PNS was transferred to 11 x 60 mm polyallomer tubes, 600 μl of ice-cold 85% sucrose in TNE was added and solutions were mixed for 5 s at 1400 rpm to adjust to a final sucrose concentration of 73%. Lysates were overlaid with 1.8 ml of ice-cold 65% sucrose in TNE and finally with 600 μl of 10% (w/v) sucrose in TNE. The gradients were centrifuged at 4°C for 16 h at 135,000 g and split into 10 aliquots of 300 μl each. Samples were denatured by adding 5x Laemmli buffer for 5 min at 95°C and 50 μl of each fraction was resolved on a 10% polyacrylamide SDS-PAGE and analyzed for protein association to membranes.

### Quantitative RT-PCR

Isolation of total RNA from cell monolayers was performed using the RNeasy Plus Mini kit according to the manufacturer’s protocol. For strand-specific qRT-PCR, 250 ng of RNA was used to synthesize cDNA with the RevertAid Reverse Transcriptase with 5 pmol of oligo(dT) as well as each tag-primer for vRNA, cRNA and mRNA as published previously [50]. Isolation of RNA from released IAV particles was performed by using TRIzol LS reagent according to the manufacturer’s protocol. 100 ng of extracted RNA were used to synthesize cDNA with the Revert AID H Minus Reverse Transcriptase and IAV universal-12 primers according to the manufacturer’s protocol. Isolation of RNA from immunoprecipitated M1 was performed by using peqGOLD TriFast reagent according to the manufacturer’s protocol. 50 ng RNA were used to synthesize cDNA with random hexamer primers. Primer sequences are listed in S1 Table. qRT-PCR reactions were performed using Brilliant III SYBR Green QPCR Master Mix following the manufacturer’s instructions. Ct values obtained from strand-specific qRT-PCR were normalized to GAPDH expression levels and quantified by using the ΔΔCt method. IAV segment incorporation was analyzed by normalization to the amount of infectious particles (PFU) as determined by plaque assay or the total amount of particles produced (HAU/PFU) as determined by hemagglutination assay.

### Indirect immunofluorescence

A549 cells were seeded in 24-well plates onto 12 mm coverslips, infected and fixed at the indicated time points with 4% Paraformaldehyde (Formaldehyde) in PBS. After 15–20 min cells were washed twice-thrice with PBS. Permeabilization was performed with 0.1%-0.2% Triton X-100 for 15 min with subtle shaking and subsequent washing (twice-thrice) with PBS. Blocking was performed with 3%-5% BSA for 1h at room temperature or o/n at 4°C with gentle shaking. Coverslips with diluted primary antibodies were incubated for 1–2 h at room temperature. Secondary antibodies Alexa 488-, Alexa 568- or Alexa 647-conjugated were used for visualization. DAPI was added in the last washing step for 10 minutes and cells on coverslips were mounted on slides using fluorescence mounting medium. Slides were analyzed using Zeiss Confocal LSM800 microscope using Zen Lite system with the Plan-Apochromat 63x/1.40 Oil DIC M27 objective and AiryScan GaAsP detector (1AU). Pictures were prepared with Fiji/ImageJ software version 1.51n.

### STORM

Samples were prepared for dSTORM as recommended by the Nikon N-STORM sample preparation protocol for immune staining using the reporter-only method with conventional antibodies. Cells were incubated on ice for 10 minutes, washed with ice cold PBS and stained for lipid rafts with Cholera Toxin B subunit Alexa 647 conjugate (1:100, Invitrogen), for 10 minutes on ice. Cells were washed with ice cold PBS, before fixing with ice cold 4% (v/v) paraformaldehyde and 0.1% (v/v) glutaraldehyde in PBS for 10 min. Samples were washed with PBS and reactive groups were quenched with 0.1 M glycine in PBS for 7 min. Cells were rinsed with PBS before permeabilization with 0.2% (v/v) Triton X-100 in PBS for 15 min. Samples were washed with PBS, blocked overnight with 10% (w/v) BSA in PBS and incubated for 60 min with 5% (w/v) BSA in PBS containing antibodies against the viral M1 protein (1:500; BioRad). Samples were rinsed with 1% (w/v) BSA in PBS. M1 primary antibodies were detected using goat anti-mouse Alexa 568 (1:500; Invitrogen) and Cholera Toxin B subunit was copatched with rabbit anti-Cholera Toxin serum (1:100, Sigma Aldrich) for 60 minutes. Samples were washed with PBS and post-fixed with 4% (v/v) paraformaldehyde and 0.1% (v/v) glutaraldehyde in PBS for 10 min.

Sample imaging was performed with the N-STORM Ti-LAPP Ti laser application system of the inverted microscope system Nikon Eclipse Ti-E, equipped with a high numerical (1.49 NA) oil-immersion HP CFI Apo TIRF 100xH objective and the TI-LA-NS N-STORM module. Fluorophores were activated with the Nikon LU-NV Series Laser Unit with the TIRF/PA LU controller B (Model: LU-TCB) containing four laser lines combined into a single optical fiber. 20 mW for 405 nm, 70 mW for 568 nm and 125 mW for 647 were measured as nominal output. For an increased laser intensity at the center of the field, the laser-TIRF illuminator was used with a beam-focusing lens. Fluorescence signals were captured by the high-resolution back-lit electron multiplying CCD camera system iXon DU-897 X-10622 Ultra EMCCD Andor with an EM Gain of 17 MHz at 16-bit and the multi-color continuous activation mode with the laser line combinations 405nm/561nm and 405nm/647nm and the activated focus stabilizing system (perfect focus system, PFS). 20,000 frames per image were taken. The imaging buffer comprised PBS supplemented with 1% (w/v) glucose, 6 nM 2-mercaptoethylamine, 0.5% (v/v) ß-mercaptoethanol (99%), 0.5% (v/v) cyclooctatetraene (98%), 40 μg/ml glucose oxidase and 3.2 μg/ml catalase.

Image reconstruction was calculated with NIS-Element Advanced Research–Imaging software (V5.11.03) using algorithms for molecule identification and drift correction.

### Electron microscopy

A549 cells were seeded in 6-cm dishes, infected and fixed in 2.5% glutaraldehyde in D-PBS [pH 7.2], post-fixed in 1% osmium tetroxide for 1 h at room temperature, blocked stained with 0.5% uranyl acetate, dehydrated in alcohol and embedded in epon. 60-nm ultrathin sections were cut and counterstained with uranyl acetate and lead. Images of selected areas were documented with Olympus Veleta 4k CCD camera or with ditabis imaging plates. Pictures were taken from different cells with different magnifications using FEI-Tecnai 12 120 kV Transmission Electron Microscope. For virus quantification, pictures were taken randomly from 10 different infected cells with the same size magnification. A total of 192 virus particles each for WSN wild type and mutant were numbered randomly and measured by their length and width using Fiji/ImageJ version 1.51n.

### Immunoprecipitation

A549 cells were infected with 5 MOI WSN WT or M1 Y132A for 9 h and lysed with TLB buffer (20mM Tris pH 7.4, 137mM NaCl, 10% glycerol, 1% Triton X 100, 2mM EDTA, 50 mM glycerol 2-phosphate, 20 mM sodium-pyrophosphate; pH 8.3) containing protease and phosphatase inhibitors. Lysates were cleared by centrifugation and supernatants were incubated o/n at 4°C with M1 antibodies coupled to protein G-conjugated agarose. Complexes were washed three times with lysis buffer and resolved by SDS PAGE with subsequent electrotransfer onto nitrocellulose membranes as described above.

### Mass spectrometry

HEK293T cells were transfected with pEBG vectors expressing GST-tagged WSN M1 WT protein (50 μg) using Lipofectamine 2000 and Opti-MEM. After 48h, transfected cells were infected with WSN WT with MOI 3. Glutathione Sepharose 4B beads (GE Healthcare) were washed 3x at 500 x g for 2 min with one volume of ice-cold RIPA lysis buffer. After the last washing step, lysis buffer was added to have a 50% slurry. At 7 and 9 hpi, all dishes were washed twice in ice cold HBS buffer (20 mM HEPES-NaOH pH 7.5, 100 mM NaCl). 1 ml ice cold HBS was added to dishes, the monolayer was scraped and transferred to 2 ml reaction tubes. Cells were pelleted at 4ºC by centrifugation at 2000 rpm for 3 min. Supernatants were removed and RIPA buffer supplemented with protease and phosphatase inhibitor cocktails was added. In the case of the dephosphorylated control, RIPA buffer was only supplemented with protease inhibitor cocktail. To remove cell debris, protein lysates were centrifuged at 20000 x g for 15 min at 4ºC. Cleared lysates were transferred to new reaction tubes and protein concentration was measured by Bradford reactive using BSA standard series (0.25–1.0 mg). Approximately 4 mg of total lysate were used for GST pull-down with 80 μl of 50% slurry Glutathione Sepharose 4B beads. 100 μl of the cleared lysate was reserved as input sample. Interaction between the beads and the lysate was allowed for 4 h in rotation at 15 rpm. Afterwards, tubes were centrifuged at 500 x g for 2 min. Supernatants were removed and beads were washed 4x with one volume of RIPA buffer supplemented with respective inhibitor cocktails. For the dephosphorylated control, 800 U Lambda protein phosphatase were consecutively added to the beads and incubated at 30ºC for 30 min. 40 μl of 1x sample buffer was added to all samples and cooked at 95ºC for 3 min. Afterwards, samples were separated in 10% SDS-PAGE followed by Commassie blue staining. Gel bands corresponding to GST-M1 were cut from the gel and kept at -80ºC until mass spectrometry analysis.

Gel bands were washed and subjected to tryptic digestion with additional reduction and alkylation. To that end, 30–50 μl 20 mM DTT in 50 mM NH_4_HCO_3_ were added to the destained and dried gel piece, and proteins were reduced for 1h at room temperature with shaking. DTT-solution was removed, 50 μl acetonitrile was added and the gel was dried in a speedvac. Alkylation proceeded with 30 μl iodoacetamide (50 mM in 50 mM NH_4_HCO_3_) for 30 min in the dark at room temperature with shaking. The solution was removed, the gel was dried as before and subsequently incubated with 30 μl DTT-solution for 15 min. The gel piece was dried again for tryptic digestion and peptide extraction. Phosphopeptide enrichment was performed according to the instructions of the manufacturer using the Titansphere Phos-TiO Kit (GL Sciences). Peptide solutions were dried and dissolved in 5 μl 0.1% formic acid containing 5% acetonitrile. Reversed-phase liquid chromatography coupled to high-definition mass spectrometry was performed using Synapt G2 Si mass spectrometer with M-Class UPLC (Waters Corp.; 30 min gradient, solvent system 100% water versus 100% acetonitrile, both containing 0.1% formic acid; flow rate 0.3 μl/min; trap column V/M Symmetry C18 100 Å 5 μm, 180 μm x 20 mm; reversed phase column HSS T3 1.8 μm 75 μm x 200 mm; 1 to 4.5 μl injection volume). Data were analysed using ProteinLynx Global Server (Waters Corp.).

Mass spectrometry of M1 isolated from viral particles was performed at the Quantitative Proteomics & Proteome Center Tuebingen. Virus particles were concentrated from supernatants of MDCKII cells infected with 0.0001 MOI of WT WSN 72 hpi. Virus particles were purified over a sucrose cushion (20% in PBS) and centrifuged at 14,000 rpm for 90 min at 4°C. Supernatants were withdrawn and virus pellets were lysed in RIPA buffer. Protein concentration was determined by Bradford assay and samples were separated by SDS PAGE followed by gel fixation and staining with coomassie. M1 bands were cut, proteins were trypsin digested and LC-MS/MS analysis was performed on a Proxeon Easy-nLC coupled to a QExactice mass spectrometer (method: 60 min, Top7, HCD). Data were processed using MaxQuant software (version 1.5.2.8. with integrated Andromeda search engine). The spectra were searched against a Homo sapiens and Influenza A Uniprot database and against the sequence of WSN M1. The data were processed with a setting of 1% for the FDR (False Discovery Rate), i.e. with an estimation that 1% of all identifications are false positive. The PEP (Posterior Error Probability [51]) was calculated for each peptide.

### Molecular dynamics simulations

The three-dimensional structure of M1 (M1, sequence MSLLTEVETYVLSIVPSGPLKAEIAQRLEDVFAGKNTDLEVLMEWLKTRPILSPLTKGILGFVFTLTVPSERGLQRRRFVQNALNGNRDPNNMDKAVKLYSKLKSEITFHGAKEIALSYSAGALASCMGLIYNRMGAVTTEVAFGLVCATCEQIADSQHRSHRQM) has been predicted by *YASARA*'s homology modeling experiment (Version 17.12.24.W.64) set at best modeling speed (slow). Solved crystal structures of Influenza A virus matrix protein M1 was identified as best suited templates (5v8a.pdb, monomeric structure), (5CQE.pdb, homodimeric structure) and (4IVJ.pdb, multimeric M1). The target residues are aligned to template residues based on sequence identity of 100.0% and the missing loops (up to 4) had to be modeled. The overall quality Z-score of all three models improved to 0.783–1.016 the minimization which can be regarded as “optimal” for all models (NOTE: the outermost residues contained in N- and C-terminal unaligned tails of models have been excluded from Z-score calculations). All further simulations were conducted with the homodimeric structure model based on 5CQE.pdb. The resulting model was named M1. Residue Y132 was mutated to Y132A and Y132F. Further, Y132 was phosphorylated resulting in Y132-P. All models were subjected to 15 nsec all-atoms-mobile MD-simulations. After this simulation time Y132A and Y132F were in equilibrium. However, for Y132-P the simulation was extended to 100 nsec to fully relax the respective structure. The mean structures for all models were computed and compared. The Poisson-Bolzmann dielectric constants was calculated for the Y132-P-M1-dimer by using atomic partial changes using PyMol version 2.2.3.

### Native PAGE

A549 cells were infected with 1 MOI of WSN WT or M1 Y132A for 16 h. Cells were lysed in native PAGE lysis buffer (50 mM Tris-HCl pH 8, 1% NP40, 150 mM NaCl), vortexed and centrifuged at 15,000 rpm at 4°C for 10 min. Protein concentrations were determined by standard Bradford assay and 2x sample buffer (125 mM Tris-HCl pH 6.8,30% glycerol, 0.1% bromphenol blue) was added. Native gels (7.5% acrylamide/bis 29:1, 0.375 M Tris-HCl pH 8.8, APS, TEMED) were pre-run for 30 min at constant current with upper chamber (25 mM Tris-HCl pH 8.4, 192 mM glycine, 1% DOC) and lower chamber buffers (25 mM Tris-HCl pH 8.4, 192 mM glycine) before sample loading. Following sample electrophoresis [52], standard western blot was performed as described above.

### Animal experiments

Virus stocks were diluted in Opti-MEM medium containing 0.3% BSA (v/w). For infection, 8 to 10 weeks old hMx-tg mice were anesthetized with a mixture of ketamine (100 mg per g body weight) and xylazine (5 mg per g body weight) administered intraperitoneally and subsequently inoculated intranasally with 40 μl of the indicated virus dose. Throughout the course of the experiment mice were monitored daily and if severe illness or weight loss of >25% occurred, mice were sacrificed by cervical dislocation. To determine organ titers, mice were euthanized at 3 dpi and indicated organs were harvested. Organs were homogenized in 1 ml PBS by three subsequent rounds of mechanical treatment for 25 s each at 6.5 ms^-1^. Tissue debris was removed by centrifuging homogenates for 5 min at 5000 rpm at 4°C and subsequently stored at -80°C for further processing. Ten-fold serial dilutions of homogenized organ samples were prepared to determine viral organ titers by plaque assay on MDCK II cells.

### Quantification and statistical analysis

Analysis of statistical significance and generation of graphs was performed by using GraphPad PRISM version 6.01. Particular tests used and sample sizes can be found in the respective figure legends. Band intensities in western blots were analyzed by using Image Studio version 5.2 and final editing of TEM, STORM and immunofluorescence pictures was performed by using Fiji/ImageJ version 1.51n. For quantification of plaque sizes, Adobe Photoshop CS6 Extended version 13.0 x64 was used. For the design and editing of final figures of molecular dynamics simulations, Chimera 1.12 was used.

## Supporting information

S1 Fig M1 Y132 is phosphorylated by JAK2 at late stages of virus replication. **A)** Mass spectrometry was performed on overexpressed GST-M1 isolated from WSN-infected A549 cells (MOI 5; 7 and 9 hpi). Depicted are the gas phase fragment ions detected for phosphorylated and carbamidomethylated peptide REITFHGAKEIALSYSAGALACCMGLIYNR (m/z 3452.631) in data-independent mass spectrometry (MSe) using Synapt G2 Si. **B)** Plaque sizes of WSN WT, M1 Y10F and M1 Y132A were quantified from neutral red-stained dilutions of standard plaque assays by using Adobe Photoshop ruler tool. Plaques (n = 30) were randomly selected and measured in diameter. Results are depicted as relative plaque size compared to WT ±SD. Statistical significance was analyzed by one-way Anova followed by Dunn’s multiple comparisons test. **C)** Quantification of viral protein expression in WSN WT versus M1 Y10F infection (see Fig 1E). Densitometric analyses of band intensities were performed by using Image Studio version 5.2 and are depicted as n-fold expression ±SD of three independent experiments. **D)** *in vitro* phosphorylation of recombinant M1 WT, M1 Y132A, M1 Y132F or GST alone by recombinant JAK2. Tyrosine phosphorylation was analyzed by Western blot. Blots are representative of two independent experiments.
(TIF)

S2 Fig M1 Y132A is efficiently translocated into the nucleus to fulfil its nuclear functions. **A)** WSN M1 WT was modelled based on the solved and refined crystal structure (pdb: 5v8a). Surface representation is colored by the electrostatic potential calculated by Poisson-Bolzman with positive in blue and negative in red. Localization of Y132 is indicated in red, positions of the nuclear localization signal in yellow and of the nuclear export signal in purple. Localization of positively charged arginine triplet (R76/77/78) is highlighted in green. **B-D)** A549 cells were infected with WSN WT or M1 Y132A (MOI 5). **B, C)** Cells were fixed 5 **(B)** or 7 hpi **(C)** and analyzed for M1 localization (red) by indirect immunofluorescence. NP (green) was used as marker for the localization of vRNPs and nuclei were stained by using DAPI (blue). Pictures were taken with LSM800 confocal microscope and analyzed with Fiji/ImageJ version 1.51n. Scale bar: 10 μm. **D)** Total RNA was isolated 5, 6 and 7 hpi and different RNA species produced for the NA segment were quantified by qRT-PCR. Depicted are RNA copies/cell ±SD of one representative of two experiments.
(TIF)

S3 Fig M1 Y132A aggregates do not co-localize with membranous organelles. **A-D)** A549 cells were infected with WSN WT or M1 Y132A (MOI 5) for 9 h. Cells were fixed and analyzed for WT M1 and M1 Y132A localization (green) by indirect immunofluorescence. **(A)** GM130 (purple) was used as marker for cis-Golgi, **(B)** TGN46 (purple) as marker for trans-Golgi, **(C)** Calnexin (purple) as marker for ER and **(D)** EEA1 (purple) as marker for early endosomes. Nuclei were stained by using DAPI (blue). Pictures were taken with LSM800 confocal microscope and analyzed with Fiji/ImageJ version 1.51n. Scale bar: 10 μm. Pictures are representatives of two-four independent experiments.
(TIF)

S4 Fig Presence of M1 Y132A induces changes in IAV budozones. **A, B)** A549 cells were infected with WSN WT or M1 Y132A (MOI 5) for 9 h. Cells were fixed with 2.5% glutaraldehyde and proceeded for electron microscopy analysis. Samples were analyzed with a 120 kV FEI-Tecnai 12 electron microscope and Fiji/ImageJ version 1.51n. **A)** Cell morphological changes upon WSN WT (panels b and d) or M1 Y132A (panels c and e) infection. Mock-infected A549 were used as control (panel a). Black arrows mark presence of stress fibers and lamellipodia. Scale bar: 1 μm. **B)** Virus particles of WSN WT (upper row) or M1 Y132A (bottom row) at different steps of the budding event. For better visualization, different steps were arbitrarily assigned numbers I-IV. (I) nascent buds can be observed as prominent bulks of the plasma membrane with high electron density due to the presence of M1 in the inner leaflet of the lipid bilayer; (II) particles in more progressed stages of assembly are observed with constricted necks at the rear end of the budding particle as a result of M1 polymerization forming the matrix lattice; (III) particles where the visible neck of the particle is completely closed since the M1 concentration has reached the protein threshold; (IV) virus particles fully assembled but not yet cleaved from the plasma membrane, and (V) finally pinched-off particles. Scale bar: 100 nm. **C)** Recombinant GST-tagged M1 proteins carrying the WT sequence or phospho-substitutions were incubated with LUVs (1:150 protein:LUVs) for 30 min, followed by centrifugation to test for co-precipitation. GST alone and incubation without liposomes were used as controls. Results of densitometric analyses are shown at the bottom. S = supernatant, P = pellet. Blots are representative of two independent experiments.
(TIF)

S5 Fig Phosphorylation of M1 tyrosine 132 is needed for efficient vRNP incorporation into budding particles. MDCKII were infected with 0.001 MOI of WSN WT or M1 Y132A. 48 h p.i. virus-containing supernatants were harvested, infectious titers were determined by standard plaque assays and HAU were analyzed by hemagglutination assays. Incorporation of vRNAs into viral particles was analyzed by qRT-PCR and normalized to the amount of infectious particles (left) or the total amount of particles (HAU/pfu; right). vRNA levels of WSN WT particles were arbitrarily set to 1. Depicted are mean vRNA incorporation levels ±SD of three independent experiments.
(TIF)

S1 Table Primers used in the study. (DOCX)

## References

[1] Keating JA, Striker R. Phosphorylation events during viral infections provide potential therapeutic targets. Rev Med Virol 2012 5;22(3):166–181. 10.1002/rmv.722 22113983 PMC3334462

[2] Kordyukova LV, Shtykova EV, Baratova LA, Svergun DI, Batishchev OV. Matrix proteins of enveloped viruses: a case study of Influenza A virus M1 protein. J Biomol Struct Dyn 2018 2 13:1–20. 10.1080/07391102.2018.1436089 29388479

[3] Nayak DP, Balogun RA, Yamada H, Zhou ZH, Barman S. Influenza virus morphogenesis and budding. Virus Res 2009 8;143(2):147–161. 10.1016/j.virusres.2009.05.010 19481124 PMC2730999

[4] Helenius A. Unpacking the incoming influenza virus. Cell 1992 5 15;69(4):577–578. 10.1016/0092-8674(92)90219-3 1375129

[5] Fontana J, Steven AC. At low pH, influenza virus matrix protein M1 undergoes a conformational change prior to dissociating from the membrane. J Virol 2013 5;87(10):5621–5628. 10.1128/JVI.00276-13 23468509 PMC3648175

[6] Martin K, Helenius A. Nuclear transport of influenza virus ribonucleoproteins: the viral matrix protein (M1) promotes export and inhibits import. Cell 1991 10 4;67(1):117–130. 10.1016/0092-8674(91)90576-k 1913813

[7] Brunotte L, Flies J, Bolte H, Reuther P, Vreede F, Schwemmle M. The nuclear export protein of H5N1 influenza A viruses recruits Matrix 1 (M1) protein to the viral ribonucleoprotein to mediate nuclear export. J Biol Chem 2014 7 18;289(29):20067–20077. 10.1074/jbc.M114.569178 24891509 PMC4106323

[8] Watanabe K, Handa H, Mizumoto K, Nagata K. Mechanism for inhibition of influenza virus RNA polymerase activity by matrix protein. J Virol 1996 1;70(1):241–247. 10.1128/JVI.70.1.241-247.1996 8523532 PMC189810

[9] Hutchinson EC, Denham EM, Thomas B, Trudgian DC, Hester SS, Ridlova G, et al Mapping the phosphoproteome of influenza A and B viruses by mass spectrometry. PLoS Pathog 2012;8(11):e1002993 10.1371/journal.ppat.1002993 23144613 PMC3493474

[10] Klemm C, Boergeling Y, Ludwig S, Ehrhardt C. Immunomodulatory Nonstructural Proteins of Influenza A Viruses. Trends Microbiol 2018 7;26(7):624–636. 10.1016/j.tim.2017.12.006 29373257

[11] Weber A, Dam S, Saul VV, Kuznetsova I, Muller C, Fritz-Wolf K, et al Phosphoproteome analysis of cells infected with adapted and non-adapted influenza A virus reveals novel pro- and antiviral signaling networks. J Virol 2019 4 17. 10.1128/JVI.00528-19 PMC6580974 30996098

[12] Wang S, Zhao Z, Bi Y, Sun L, Liu X, Liu W. Tyrosine 132 phosphorylation of influenza A virus M1 protein is crucial for virus replication by controlling the nuclear import of M1. J Virol 2013 6;87(11):6182–6191. 10.1128/JVI.03024-12 23536660 PMC3648105

[13] Harris A, Cardone G, Winkler DC, Heymann JB, Brecher M, White JM, et al Influenza virus pleiomorphy characterized by cryoelectron tomography. Proc Natl Acad Sci U S A 2006 12 12;103(50):19123–19127. 10.1073/pnas.0607614103 17146053 PMC1748186

[14] Leser GP, Lamb RA. Lateral Organization of Influenza Virus Proteins in the Budozone Region of the Plasma Membrane. J Virol 2017 4 13;91(9): 10.1128/JVI.02104-16 Print 2017 May 1. 28202765 PMC5391459

[15] Dubois J, Terrier O, Rosa-Calatrava M. Influenza viruses and mRNA splicing: doing more with less. MBio 2014 5 13;5(3):e00070–14. 10.1128/mBio.00070-14 24825008 PMC4030477

[16] Konig R, Stertz S, Zhou Y, Inoue A, Hoffmann HH, Bhattacharyya S, et al Human host factors required for influenza virus replication. Nature 2010 2 11;463(7282):813–817. 10.1038/nature08699 20027183 PMC2862546

[17] Han J, Perez JT, Chen C, Li Y, Benitez A, Kandasamy M, et al Genome-wide CRISPR/Cas9 Screen Identifies Host Factors Essential for Influenza Virus Replication. Cell Rep 2018 4 10;23(2):596–607. 10.1016/j.celrep.2018.03.045 29642015 PMC5939577

[18] Das SC, Watanabe S, Hatta M, Noda T, Neumann G, Ozawa M, et al The highly conserved arginine residues at positions 76 through 78 of influenza A virus matrix protein M1 play an important role in viral replication by affecting the intracellular localization of M1. J Virol 2012 2;86(3):1522–1530. 10.1128/JVI.06230-11 22090133 PMC3264381

[19] Kerviel A, Dash S, Moncorge O, Panthu B, Prchal J, Decimo D, et al Involvement of an Arginine Triplet in M1 Matrix Protein Interaction with Membranes and in M1 Recruitment into Virus-Like Particles of the Influenza A(H1N1)pdm09 Virus. PLoS One 2016 11 4;11(11):e0165421 10.1371/journal.pone.0165421 27814373 PMC5096668

[20] Sha B, Luo M. Structure of a bifunctional membrane-RNA binding protein, influenza virus matrix protein M1. Nat Struct Biol 1997 3;4(3):239–244. 10.1038/nsb0397-239 9164466

[21] Dadonaite B, Vijayakrishnan S, Fodor E, Bhella D, Hutchinson EC. Filamentous influenza viruses. J Gen Virol 2016 8;97(8):1755–1764. 10.1099/jgv.0.000535 27365089 PMC5935222

[22] Ma H, Kien F, Maniere M, Zhang Y, Lagarde N, Tse KS, et al Human annexin A6 interacts with influenza a virus protein M2 and negatively modulates infection. J Virol 2012 2;86(3):1789–1801. 10.1128/JVI.06003-11 22114333 PMC3264383

[23] Gomez-Puertas P, Albo C, Perez-Pastrana E, Vivo A, Portela A. Influenza virus matrix protein is the major driving force in virus budding. J Virol 2000 12;74(24):11538–11547. 10.1128/jvi.74.24.11538-11547.2000 11090151 PMC112434

[24] Chen BJ, Leser GP, Jackson D, Lamb RA. The influenza virus M2 protein cytoplasmic tail interacts with the M1 protein and influences virus assembly at the site of virus budding. J Virol 2008 10;82(20):10059–10070. 10.1128/JVI.01184-08 18701586 PMC2566248

[25] McCown MF, Pekosz A. Distinct domains of the influenza a virus M2 protein cytoplasmic tail mediate binding to the M1 protein and facilitate infectious virus production. J Virol 2006 8;80(16):8178–8189. 10.1128/JVI.00627-06 16873274 PMC1563831

[26] Zhang J, Pekosz A, Lamb RA. Influenza virus assembly and lipid raft microdomains: a role for the cytoplasmic tails of the spike glycoproteins. J Virol 2000 5;74(10):4634–4644. 10.1128/jvi.74.10.4634-4644.2000 10775599 PMC111983

[27] Brown DA, Rose JK. Sorting of GPI-anchored proteins to glycolipid-enriched membrane subdomains during transport to the apical cell surface. Cell 1992 2 7;68(3):533–544. 10.1016/0092-8674(92)90189-j 1531449

[28] Scheiffele P, Rietveld A, Wilk T, Simons K. Influenza viruses select ordered lipid domains during budding from the plasma membrane. J Biol Chem 1999 1 22;274(4):2038–2044. 10.1074/jbc.274.4.2038 9890962

[29] Magee AI, Parmryd I. Detergent-resistant membranes and the protein composition of lipid rafts. Genome Biol 2003;4(11):234-2003-4-11-234. Epub 2003 Oct 27. 10.1186/gb-2003-4-11-234 PMC329107 14611651

[30] Pike LJ. Growth factor receptors, lipid rafts and caveolae: an evolving story. Biochim Biophys Acta 2005 12 30;1746(3):260–273. 10.1016/j.bbamcr.2005.05.005 15951036

[31] Wang D, Harmon A, Jin J, Francis DH, Christopher-Hennings J, Nelson E, et al The lack of an inherent membrane targeting signal is responsible for the failure of the matrix (M1) protein of influenza A virus to bud into virus-like particles. J Virol 2010 5;84(9):4673–4681. 10.1128/JVI.02306-09 20181696 PMC2863757

[32] Hilsch M, Goldenbogen B, Sieben C, Hofer CT, Rabe JP, Klipp E, et al Influenza A matrix protein M1 multimerizes upon binding to lipid membranes. Biophys J 2014 8 19;107(4):912–923. 10.1016/j.bpj.2014.06.042 25140426 PMC4142230

[33] Xie H, Lin Z, Mosier PD, Desai UR, Gao Y. The compensatory G88R change is essential in restoring the normal functions of influenza A/WSN/33 virus matrix protein 1 with a disrupted nuclear localization signal. J Virol 2013 1;87(1):345–353. 10.1128/JVI.02024-12 23077315 PMC3536423

[34] Safo MK, Musayev FN, Mosier PD, Zhou Q, Xie H, Desai UR. Crystal structures of influenza A virus matrix protein M1: variations on a theme. PLoS One 2014 10 8;9(10):e109510 10.1371/journal.pone.0109510 25295515 PMC4190115

[35] Chaimayo C, Hayashi T, Underwood A, Hodges E, Takimoto T. Selective incorporation of vRNP into influenza A virions determined by its specific interaction with M1 protein. Virology 2017 5;505:23–32. 10.1016/j.virol.2017.02.008 28219018 PMC5366082

[36] Noda T, Sugita Y, Aoyama K, Hirase A, Kawakami E, Miyazawa A, et al Three-dimensional analysis of ribonucleoprotein complexes in influenza A virus. Nat Commun 2012 1 24;3:639 10.1038/ncomms1647 22273677 PMC3272569

[37] von Magnus P. Incomplete forms of influenza virus. Adv Virus Res 1954;2:59–79. 10.1016/s0065-3527(08)60529-1 13228257

[38] Deeg CM, Hassan E, Mutz P, Rheinemann L, Gotz V, Magar L, et al In vivo evasion of MxA by avian influenza viruses requires human signature in the viral nucleoprotein. J Exp Med 2017 5 1;214(5):1239–1248. 10.1084/jem.20161033 28396461 PMC5413327

[39] Behrmann I, Smyczek T, Heinrich PC, Schmitz-Van de Leur H, Komyod W, Giese B, et al Janus kinase (Jak) subcellular localization revisited: the exclusive membrane localization of endogenous Janus kinase 1 by cytokine receptor interaction uncovers the Jak.receptor complex to be equivalent to a receptor tyrosine kinase. J Biol Chem 2004 8 20;279(34):35486–35493. 10.1074/jbc.M404202200 15123646

[40] Arzt S, Baudin F, Barge A, Timmins P, Burmeister WP, Ruigrok RW. Combined results from solution studies on intact influenza virus M1 protein and from a new crystal form of its N-terminal domain show that M1 is an elongated monomer. Virology 2001 1 20;279(2):439–446. 10.1006/viro.2000.0727 11162800

[41] Baudin F, Petit I, Weissenhorn W, Ruigrok RW. In vitro dissection of the membrane and RNP binding activities of influenza virus M1 protein. Virology 2001 3 1;281(1):102–108. 10.1006/viro.2000.0804 11222100

[42] Curthoys NM, Mlodzianoski MJ, Parent M, Butler MB, Raut P, Wallace J, et al Influenza Hemagglutinin Modulates Phosphatidylinositol 4,5-Bisphosphate Membrane Clustering. Biophys J 2019 3 5;116(5):893–909. 10.1016/j.bpj.2019.01.017 30773293 PMC6400828

[43] Calder LJ, Wasilewski S, Berriman JA, Rosenthal PB. Structural organization of a filamentous influenza A virus. Proc Natl Acad Sci U S A 2010 6 8;107(23):10685–10690. 10.1073/pnas.1002123107 20498070 PMC2890793

[44] Noton SL, Medcalf E, Fisher D, Mullin AE, Elton D, Digard P. Identification of the domains of the influenza A virus M1 matrix protein required for NP binding, oligomerization and incorporation into virions. J Gen Virol 2007 8;88(Pt 8):2280–2290. 10.1099/vir.0.82809-0 17622633 PMC2884976

[45] Chen BJ, Takeda M, Lamb RA. Influenza virus hemagglutinin (H3 subtype) requires palmitoylation of its cytoplasmic tail for assembly: M1 proteins of two subtypes differ in their ability to support assembly. J Virol 2005 11;79(21):13673–13684. 10.1128/JVI.79.21.13673-13684.2005 16227287 PMC1262586

[46] Chlanda P, Mekhedov E, Waters H, Sodt A, Schwartz C, Nair V, et al Palmitoylation Contributes to Membrane Curvature in Influenza A Virus Assembly and Hemagglutinin-Mediated Membrane Fusion. J Virol 2017 10 13;91(21): 10.1128/JVI.00947-17 Print 2017 Nov 1. 28794042 PMC5640829

[47] Hutchinson EC, Curran MD, Read EK, Gog JR, Digard P. Mutational analysis of cis-acting RNA signals in segment 7 of influenza A virus. J Virol 2008 12;82(23):11869–11879. 10.1128/JVI.01634-08 18815307 PMC2583641

[48] Ye ZP, Baylor NW, Wagner RR. Transcription-inhibition and RNA-binding domains of influenza A virus matrix protein mapped with anti-idiotypic antibodies and synthetic peptides. J Virol 1989 9;63(9):3586–3594. 10.1128/JVI.63.9.3586-3594.1989 2474671 PMC250948

[49] Elster C, Larsen K, Gagnon J, Ruigrok RW, Baudin F. Influenza virus M1 protein binds to RNA through its nuclear localization signal. J Gen Virol 1997 7;78 (Pt 7)(Pt 7):1589–1596. 9225034 10.1099/0022-1317-78-7-1589

[50] Kawakami E, Watanabe T, Fujii K, Goto H, Watanabe S, Noda T, et al Strand-specific real-time RT-PCR for distinguishing influenza vRNA, cRNA, and mRNA. J Virol Methods 2011 4;173(1):1–6. 10.1016/j.jviromet.2010.12.014 21185869 PMC3049850

[51] Kall L, Storey JD, MacCoss MJ, Noble WS. Posterior error probabilities and false discovery rates: two sides of the same coin. J Proteome Res 2008 1;7(1):40–44. 10.1021/pr700739d 18052118

[52] Iwamura T, Yoneyama M, Yamaguchi K, Suhara W, Mori W, Shiota K, et al Induction of IRF-3/-7 kinase and NF-kappaB in response to double-stranded RNA and virus infection: common and unique pathways. Genes Cells 2001 4;6(4):375–388. 10.1046/j.1365-2443.2001.00426.x 11318879

