# Supplementary figures and images for: Discrete spatio-temporal regulation of tyrosine phosphorylation directs influenza A virus M1 protein towards its function in virion assembly
Source: PLoS Pathog. 2020 Aug 31;16(8):e1008775. doi: 10.1371/journal.ppat.1008775 (PMC7485975; doi:10.1371/journal.ppat.1008775)

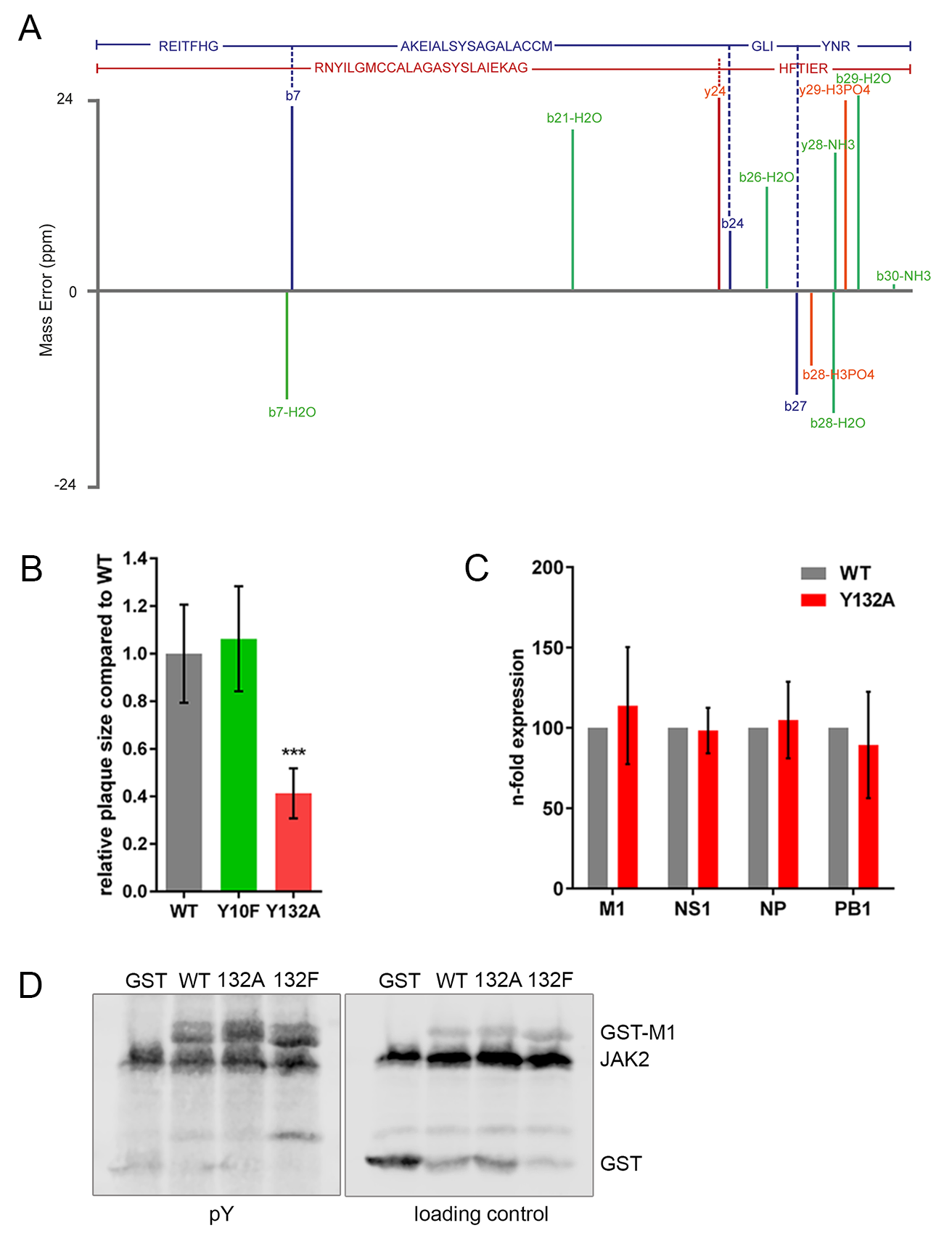

Supplement: S1 Fig — A) Mass spectrometry was performed on overexpressed GST-M1 isolated from WSN-infected A549 cells (MOI 5; 7 and 9 hpi). Depicted are the gas phase fragment ions detected for phosphorylated and carbamidomethylated peptide REITFHGAKEIALSYSAGALACCMGLIYNR (m/z 3452.631) in data-independent mass spectrometry (MSe) using Synapt G2 Si. B) Plaque sizes of WSN WT, M1 Y10F and M1 Y132A were quantified from neutral red-stained dilutions of standard plaque assays by using Adobe Photoshop ruler tool. Plaques (n = 30) were randomly selected and measured in diameter. Results are depicted as relative plaque size compared to WT ±SD. Statistical significance was analyzed by one-way Anova followed by Dunn’s multiple comparisons test. C) Quantification of viral protein expression in WSN WT versus M1 Y10F infection (see Fig 1E). Densitometric analyses of band intensities were performed by using Image Studio version 5.2 and are depicted as n-fold expression ±SD of three independent experiments. D) in vitro phosphorylation of recombinant M1 WT, M1 Y132A, M1 Y132F or GST alone by recombinant JAK2. Tyrosine phosphorylation was analyzed by Western blot. Blots are representative of two independent experiments. (TIF) [file ppat.1008775.s001.tif]

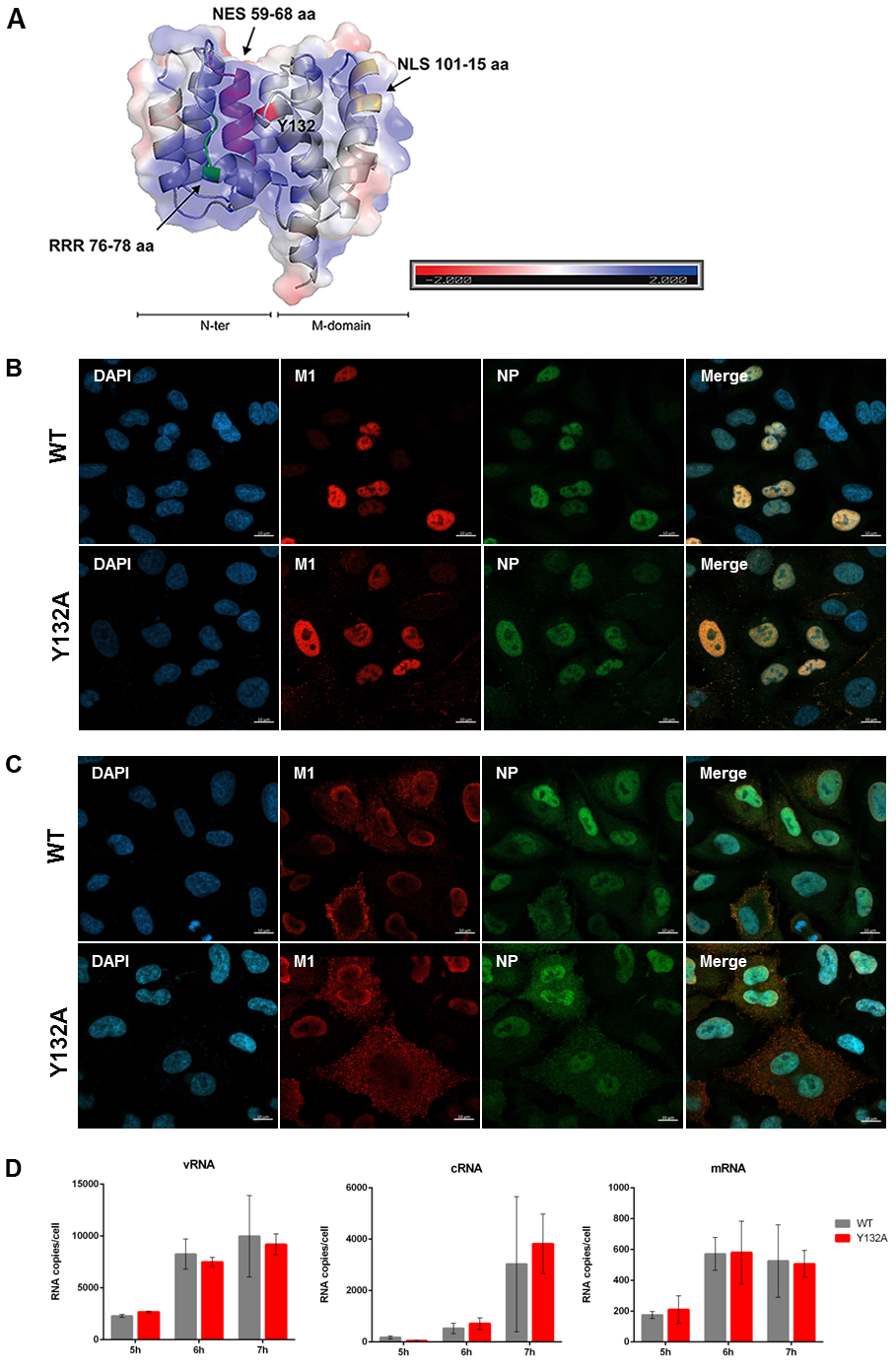

Supplement: S2 Fig — A) WSN M1 WT was modelled based on the solved and refined crystal structure (pdb: 5v8a). Surface representation is colored by the electrostatic potential calculated by Poisson-Bolzman with positive in blue and negative in red. Localization of Y132 is indicated in red, positions of the nuclear localization signal in yellow and of the nuclear export signal in purple. Localization of positively charged arginine triplet (R76/77/78) is highlighted in green. B-D) A549 cells were infected with WSN WT or M1 Y132A (MOI 5). B, C) Cells were fixed 5 (B) or 7 hpi (C) and analyzed for M1 localization (red) by indirect immunofluorescence. NP (green) was used as marker for the localization of vRNPs and nuclei were stained by using DAPI (blue). Pictures were taken with LSM800 confocal microscope and analyzed with Fiji/ImageJ version 1.51n. Scale bar: 10 μm. D) Total RNA was isolated 5, 6 and 7 hpi and different RNA species produced for the NA segment were quantified by qRT-PCR. Depicted are RNA copies/cell ±SD of one representative of two experiments. (TIF) [file ppat.1008775.s002.tif]

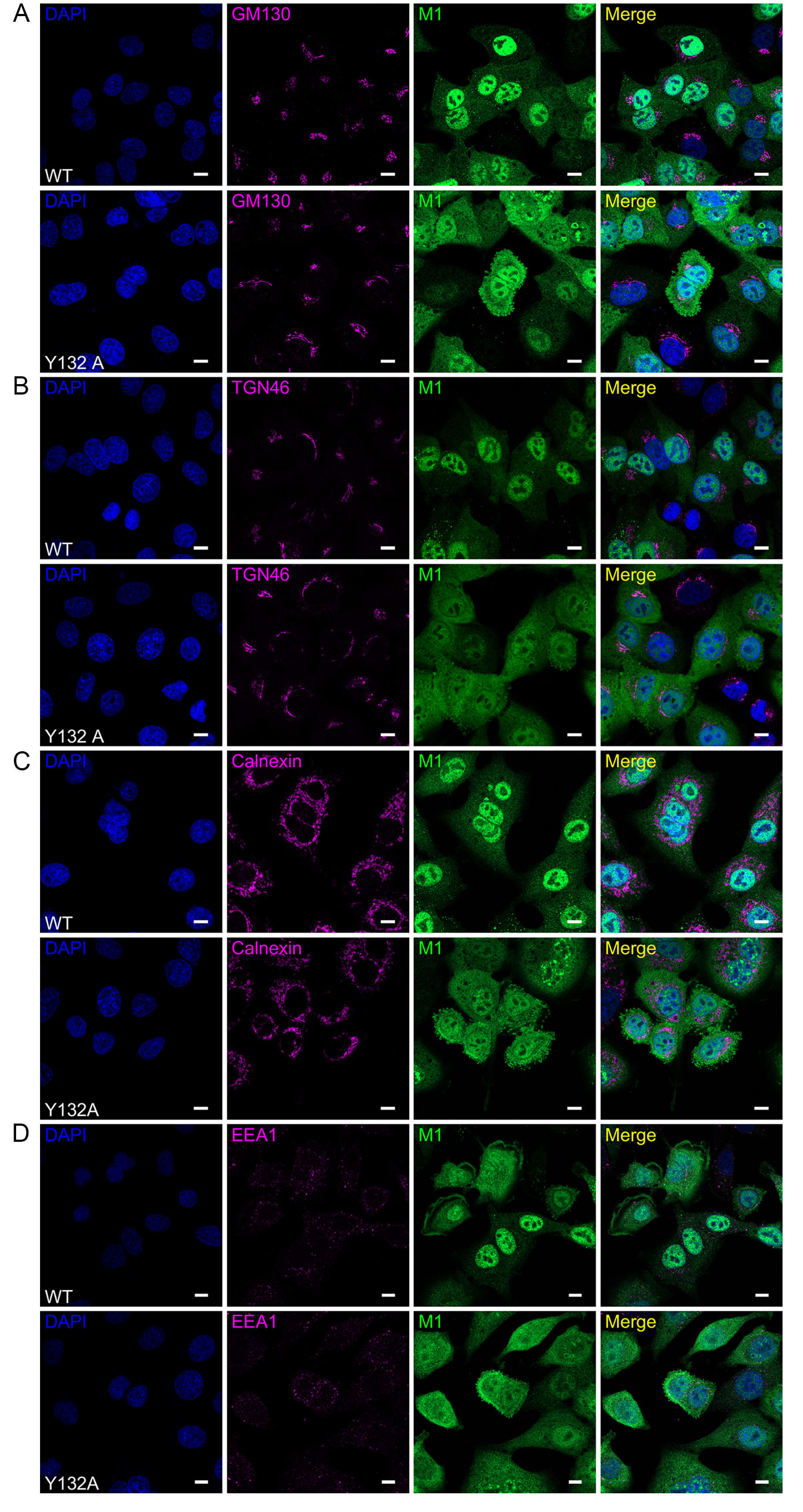

Supplement: S3 Fig — A-D) A549 cells were infected with WSN WT or M1 Y132A (MOI 5) for 9 h. Cells were fixed and analyzed for WT M1 and M1 Y132A localization (green) by indirect immunofluorescence. (A) GM130 (purple) was used as marker for cis-Golgi, (B) TGN46 (purple) as marker for trans-Golgi, (C) Calnexin (purple) as marker for ER and (D) EEA1 (purple) as marker for early endosomes. Nuclei were stained by using DAPI (blue). Pictures were taken with LSM800 confocal microscope and analyzed with Fiji/ImageJ version 1.51n. Scale bar: 10 μm. Pictures are representatives of two-four independent experiments. (TIF) [file ppat.1008775.s003.tif]

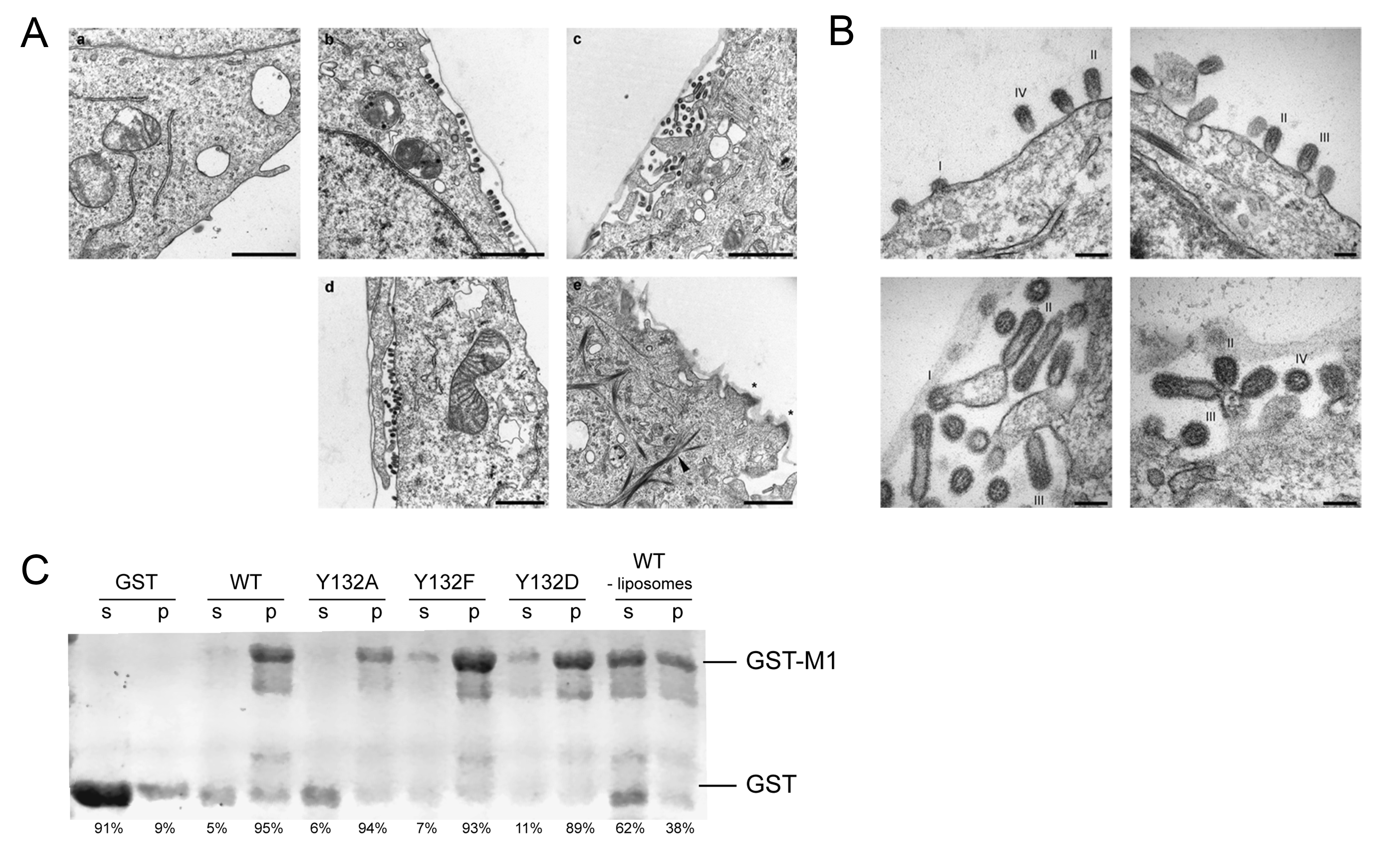

Supplement: S4 Fig — A, B) A549 cells were infected with WSN WT or M1 Y132A (MOI 5) for 9 h. Cells were fixed with 2.5% glutaraldehyde and proceeded for electron microscopy analysis. Samples were analyzed with a 120 kV FEI-Tecnai 12 electron microscope and Fiji/ImageJ version 1.51n. A) Cell morphological changes upon WSN WT (panels b and d) or M1 Y132A (panels c and e) infection. Mock-infected A549 were used as control (panel a). Black arrows mark presence of stress fibers and lamellipodia. Scale bar: 1 μm. B) Virus particles of WSN WT (upper row) or M1 Y132A (bottom row) at different steps of the budding event. For better visualization, different steps were arbitrarily assigned numbers I-IV. (I) nascent buds can be observed as prominent bulks of the plasma membrane with high electron density due to the presence of M1 in the inner leaflet of the lipid bilayer; (II) particles in more progressed stages of assembly are observed with constricted necks at the rear end of the budding particle as a result of M1 polymerization forming the matrix lattice; (III) particles where the visible neck of the particle is completely closed since the M1 concentration has reached the protein threshold; (IV) virus particles fully assembled but not yet cleaved from the plasma membrane, and (V) finally pinched-off particles. Scale bar: 100 nm. C) Recombinant GST-tagged M1 proteins carrying the WT sequence or phospho-substitutions were incubated with LUVs (1:150 protein:LUVs) for 30 min, followed by centrifugation to test for co-precipitation. GST alone and incubation without liposomes were used as controls. Results of densitometric analyses are shown at the bottom. S = supernatant, P = pellet. Blots are representative of two independent experiments. (TIF) [file ppat.1008775.s004.tif]

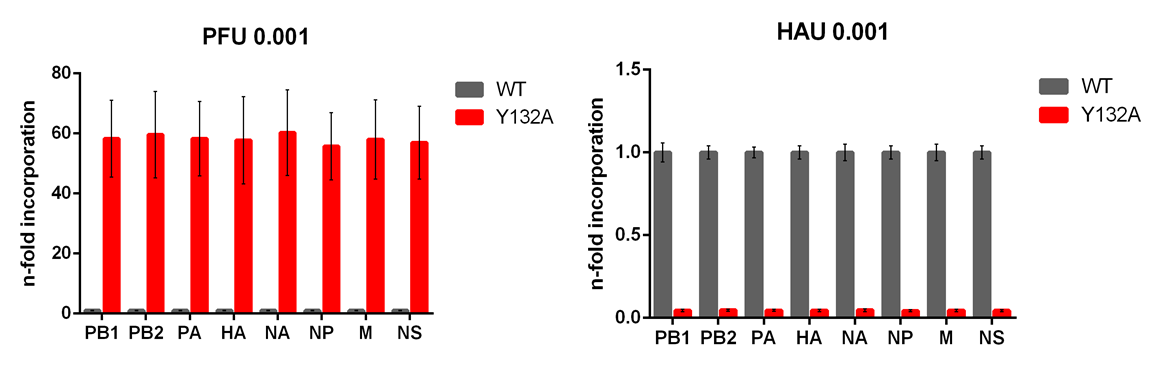

Supplement: S5 Fig — MDCKII were infected with 0.001 MOI of WSN WT or M1 Y132A. 48 h p.i. virus-containing supernatants were harvested, infectious titers were determined by standard plaque assays and HAU were analyzed by hemagglutination assays. Incorporation of vRNAs into viral particles was analyzed by qRT-PCR and normalized to the amount of infectious particles (left) or the total amount of particles (HAU/pfu; right). vRNA levels of WSN WT particles were arbitrarily set to 1. Depicted are mean vRNA incorporation levels ±SD of three independent experiments. (TIF) [file ppat.1008775.s005.tif]
