# Supplementary material for: Discrete spatio-temporal regulation of tyrosine phosphorylation directs influenza A virus M1 protein towards its function in virion assembly
Source: PLoS Pathog. 2020 Aug 31;16(8):e1008775. doi: 10.1371/journal.ppat.1008775 (PMC7485975; doi:10.1371/journal.ppat.1008775)
Supplement: S1 Table — (DOCX) [file ppat.1008775.s006.docx]

**Table S1. Primers used in the study.**

| **Use** | **Name** | **Sequence (5’ 🡪 3’)** |
| --- | --- | --- |
| Site-directed mutagenesis PCR | WSN-M1-Y10F fwd  WSN-M1-Y10F rev | CCG AGG TCG AAA CGT TCG TTC TCT CTA TCG  CGA TAG AGA GAA CGA ACG TTT CGA CCT CGG |
|  | WSN-M1-Y132A fwd  WSN-M1-Y132A rev | GTA TGG GCC TCA TAG CCA AGA GGA TGG GGG  CCC CCA TCC TGT TGG CTA TGA GGC CCA TAC |
|  | WSN-M1-Y132F fwd  WSN-M1-Y132F rev | GTA TGG GCC TCA TAT TCA ACA GGA TGG GGG  CCC CCA TCC TGT TGA ATA TGA GGC CCA TAC |
|  | WSN-M1-Y132D fwd  WSN-M1-Y132D rev | GTA TGG GCC TCA TAG ACA ACA GGA TGG GGG C  GCC CCC ATC CTG TTG TCT ATG AGG CCC ATA C |
| vRNA detection by qRT-PCR | WSN_PB1 fwd  WSN_PB1 rev | CCG ACA GAC CTA TGA CTG GAC  TAT TGT GTT GGC CAA TGC TG |
|  | WSN_PB2 fwd  WSN_PB2 rev | CGG ATC AGA CCG AGT GAT G  GGT CCA TTC CTA TTC CAC CA |
|  | WSN_PA fwd  WSN_PA rev | CTG ACC CAA GAC TTG AAC CAC  AGC ATA TCT CCT ATC TCA AGA ACA CA |
|  | WSN_HA fwd  WSN_HA rev | GCT GCA AGA CCC AAA GTA AGA  TCC GGG TTC TAG CAA GGT C |
|  | WSN_NA fwd  WSN_NA rev | ACA ACG GCA TAA TAA CTG AAA CC  CAG GTA CAT TCA GAC TCT TGT GTT C |
|  | WSN_NP fwd  WSN_NP rev | CCG GGG AAA GAT CCT AAG AA  TCC ACT TTC CAT CTA CTC TCC TG |
|  | WSN_M fwd  WSN_M rev | AAA TGG CTG GAT CGA GTG AG  GCC TGG CCT GAC TAG CAA TA |
|  | WSN_M2 fwd  WSN_M2 rev | CGA GGT CGA AAC GCC TAT CAG AAA C  CCA ATG ATA TTT GCT GCA ATG ACG AG |
|  | WSN_NS fwd  WSN_NS rev | AGC ACT CTC GGT CTG GAC AT  CCG CTC CAC TAT TTG CTT TC |
| cDNA synthesis | oligo(dT) | TTT TTT TTT TTT TTT T |
|  | Universal-12 primer | AGC AGA AAG CAG G |
|  | vRNAtag_WSNseg5_740F  vRNAtag | GGCCGTCATGGTGGCGAAT GAATGGACGGAGAACAAGGATTGC  GGCCGTCATGGTGGCGAAT |
|  | cRNAtag_WSNseg5_1565R  cRNAtag | GCTAGCTTCAGCTAGGCATC AGTAGAAACAAGGGTATTTTTCTTT  GCTAGCTTCAGCTAGGCATC |
|  | mRNAtag_WSNseg5_dTR  mRNAtag | CCAGATCGTTCGAGTCGT TTTTTTTTTTTTTTTTCTTTAATTGTC  CCAGATCGTTCGAGTCGT |
|  | vRNAtag_WSNseg6_689F  vRNAtag | GGCCGTCATGGTGGCGAAT ACCATAATGACCGATGGCCCAAGT  GGCCGTCATGGTGGCGAAT |
|  | cRNAtag_WSNseg6_1413R  cRNAtag | GCTAGCTTCAGCTAGGCATC AGTAGAAACAAGGAGTTTTTTGAAC  GCTAGCTTCAGCTAGGCATC |
|  | mRNAtag_WSNseg6_dTR  mRNAtag | CCAGATCGTTCGAGTCGT TTTTTTTTTTTTTTTTGAACAAACTAC  CCAGATCGTTCGAGTCGT |
| for qRT-PCR | WSNseg5_845R | CTCAATATGAGTGCAGACCGTGCT |
|  | WSNseg5_1466F | CGATCGTGCCCTCCTTTG |
|  | WSNseg6_839R | ACATCACTTTGCCGGTATCAGGGT |
|  | WSNseg6_1314F | TGAATAGTGATACTGTAGATTGGTCT |
|  | WSNseg6_1314F | TGAATAGTGATACTGTAGATTGGTCT |
|  | human_GAPDH_fwd  human_GAPDH_rev | GCA AAT TCC ATG GCA CCG T  GCC CCA CTT GAT TTG GAG G |
